# Whole transcriptome profiling of taste bud cells

**DOI:** 10.1038/s41598-017-07746-z

**Published:** 2017-08-08

**Authors:** Sunil K. Sukumaran, Brian C. Lewandowski, Yumei Qin, Ramana Kotha, Alexander A. Bachmanov, Robert F. Margolskee

**Affiliations:** 1 Monell Chemical Senses Center, 3500 Market Street, Philadelphia, PA 19104 USA; 2 College of Food & Biology Engineering, Zhejiang Gongshang University, Hangzhou, 310018 P.R. China

## Abstract

Analysis of single-cell RNA-Seq data can provide insights into the specific functions of individual cell types that compose complex tissues. Here, we examined gene expression in two distinct subpopulations of mouse taste cells: *Tas1r3-*expressing type II cells and physiologically identified type III cells. Our RNA-Seq libraries met high quality control standards and accurately captured differential expression of marker genes for type II (e.g. the *Tas1r* genes, *Plcb2*, *Trpm5*) and type III (e.g. *Pkd2l1*, *Ncam*, *Snap25*) taste cells. Bioinformatics analysis showed that genes regulating responses to stimuli were up-regulated in type II cells, while pathways related to neuronal function were up-regulated in type III cells. We also identified highly expressed genes and pathways associated with chemotaxis and axon guidance, providing new insights into the mechanisms underlying integration of new taste cells into the taste bud. We validated our results by immunohistochemically confirming expression of selected genes encoding synaptic (*Cplx2* and *Pclo*) and semaphorin signalling pathway (*Crmp2*, *PlexinB1*, *Fes* and *Sema4a*) components. The approach described here could provide a comprehensive map of gene expression for all taste cell subpopulations and will be particularly relevant for cell types in taste buds and other tissues that can be identified only by physiological methods.

## Introduction

Taste buds are composed of 50–100 specialized neuro-epithelial cells that have classically been divided into type I, II, and III taste cells based originally on morphology and more recently on gene expression patterns^1, 2^. Type I cells are believed to function as glia-like support cells. Type II cells, also known as receptor cells, express G protein-coupled receptors (GPCRs) for sweet, bitter, and umami stimuli. Type III cells respond to sour and high concentrations of salt through incompletely understood mechanisms; these cells are also known as pre-synaptic cells because they contain synaptic vesicles, form well defined synapses with afferent nerve fibres, and express numerous neuronal proteins.

Molecular genetic studies have led to the discovery of multiple genes encoding the receptors and downstream transduction molecules for a few of the basic tastes. They include the GPCRs responsible for sweet (*Tas1r2* + *Tas1r3*), umami (*Tas1r1* + *Tas1r3*), and bitter (*Tas2rs*) tastes, and an ion channel, the epithelial sodium channel (ENaC), that mediates one of the two salty taste transduction pathways in mammals^3–8^. In addition, genes encoding key components of the downstream signalling pathway shared by sweet, bitter, and umami tastes have been identified, including *Gnat3*, *Gnat1*, *Gnat2*, *Gna14*, *Gng13, Plcb2*, *Itpr3*, and *Trpm5* ^9–17^. Many of these discoveries were driven by methods such as differential display PCR and genetic mapping, which are limited in throughput and sensitivity compared with newer techniques such as microarrays and next-generation RNA sequencing (RNA-Seq). These whole transcriptome expression profiling technologies enable identification of practically all the genes expressed in a sample. To date, only a few such studies have been done in the taste system, using the less sensitive microarray technology and bulk taste tissue as the source of RNA^18–20^, which limits their utility because cell subtype and single-cell resolution is lacking. In addition, potentially important genes that are expressed at lower levels, or expressed in small subpopulations of cells, may be difficult to detect in expression data derived from samples containing pooled cells of different types. These disadvantages are overcome by single-cell RNA-Seq. Individual cells from a particular subpopulation of taste cells can be isolated from existing transgenic mice expressing reporters such as green fluorescent protein (GFP) under taste cell-type-specific promoters. However, there is no well-established methodology for identifying single cells from taste cell subpopulations without previously known genetic markers or distinguishing morphological features. Here we present methods that enable single-cell RNA-Seq analysis of physiologically identified cells. These techniques facilitate a more complete understanding of the genes expressed by individual taste cell types, which may provide new insights into the biological basis of taste and help answer important unresolved questions in the fields, such as the identity of the receptor(s) for sour taste and non-ENaC-mediated salt taste^21–24^.

In this study we used our single-cell RNA-Seq method to profile the transcriptomes of two subtypes of mouse taste cells: type II cells expressing *Tas1r3* (*Tas1r3*+) and type III taste cells exhibiting KCl depolarization-mediated calcium responses. We confirmed the high quality of the RNA-Seq data generated from these single taste cells using multiple quality control metrics and analysis of cell-type-specific marker gene expression. Further, we used Gene Ontology and Ingenuity Pathway Analysis (IPA) to describe and compare the pathways expressed by the type II and type III cells. Finally, we identified novel and little studied genes in our transcriptome data set that are involved in the function of synapses and in the semaphorin signalling pathway, and used immunohistochemistry to validate the expression of several of these genes in taste bud cells. Our results provide an approach for using single-cell RNA-Seq to gain an understanding of how the thousands of genes expressed by individual taste cells shape their functions and roles within the taste bud microsystem. The methodology described in this article could be used for single-cell RNA-Seq analysis of any cell type.

## Results

### Isolation of individual *Tas1r3*+ type II and physiologically-identified type III taste cells

To gain insights into what genes distinguish different types of taste cells from each other we first set out to identify two distinct taste cell populations: The *Tas1r3*-expressing subset of type II cells, and type III cells. Using dissociated taste cell preparations made from the circumvallate (CV) papillae of Tas1r3-GFP transgenic mice, Tas1r3-GFP cells (n = 9) were identified by their intrinsic fluorescence and collected manually (Supplementary Fig. S1). TAS1R3 is a key component of both the sweet and umami receptors and serves as a marker for the sweet- and umami-responsive *Tas1r3*+ subset of type II cells. Due to their intrinsic fluorescence, Tas1r3-GFP cells are readily identified and collected and can serve as a standard for comparison to other types of taste cells that are not as easily identified, such as taste cells that to date can only be reliably identified by their physiological responses to taste stimuli. These include sour-responsive cells (because it isn’t entirely clear if *all* PKD2L1-expressing cells are responsive to sour)^23, 25^, salt-responsive taste cells of both the amiloride-sensitive and amiloride-insensitive salt taste pathways^26, 27^, and the type II taste cells that respond to both bitter and salty taste stimuli^28^. To gain further insights into the nature of type III taste cells, we identified these cells by physiological methods. Unlike type I and type II taste cells, type III taste cells express both potassium leak channels and voltage-gated calcium channels and respond to depolarization with large and prolonged increases in intracellular calcium levels^29, 30^. Using fura-2 calcium imaging of single-cell preparations from the CV papillae, we identified and collected type III taste cells (n = 17) using 50 mM KCl as a depolarizing stimulus (Supplementary Fig. S1).

### Single-cell RNA-Seq of *Tas1r3*+ and type III taste cells

To obtain a sufficient amount of RNA for RNA-Seq, the mRNA population in each individual isolated taste cell was selectively amplified using the aRNA amplification method^31^. We could generate 1–5 μg of aRNA after two rounds of amplification. As is typical for single-cell aRNA amplification, the size of aRNA (200–700 bp) was much smaller than that of intact cellular mRNA and mostly represents the 3′ end of mRNAs (Supplementary Fig. S2). The aRNA was used as input for preparing un-stranded Illumina sequencing libraries, and 100-bp paired-end sequencing reads were obtained. The total number of reads per library ranged from 34 to 210 million (Supplementary Table S1). Three libraries from type III cells were removed from further analysis after quality control indicated low sequence alignment (8–18%) to the mouse reference genome. From the remaining 23 libraries, we could align 56–92% of the raw sequences to the mouse genome (Supplementary Table S1). After alignment to the genome, we counted the number of reads mapping to the set of mouse genes annotated in Gencode (release M4). In the 23 samples, 26–67% of the sequences aligned to the genome could be uniquely assigned to exons, while the others mapped to multiple locations (multi-reads) or to non-exonic regions (Supplementary Table S1). There were no significant differences in the number of total reads, genome mapped reads, or gene mapped reads between *Tas1r3*+ and type III cells, but the proportion of multi-reads was significantly lower in type III cells (Supplementary Table S2).

We normalized the gene expression values across our single-cell samples using the DESeq2 package in R^32, 33^. Of the 43,346 genes included in Gencode M4 annotation of the mouse genome assembly, 31,021 (19,474 of them protein coding) were represented by at least one count in at least one of the analysed cells; this subset of genes was used for normalization and subsequent analyses (Supplementary Table S3). To separate genes with negligibly low expression from genes with meaningful expression, we used several thresholds ranging from 1 to 50 normalized counts. Across the single-cell transcriptome libraries, the number of genes with expression levels exceeding these thresholds ranged from ~5,000 to ~16,000 (Supplementary Table S1). On average, *Tas1r3*+ cells expressed significantly more genes than did type III cells (Supplementary Table S2).

**Figure 1:**
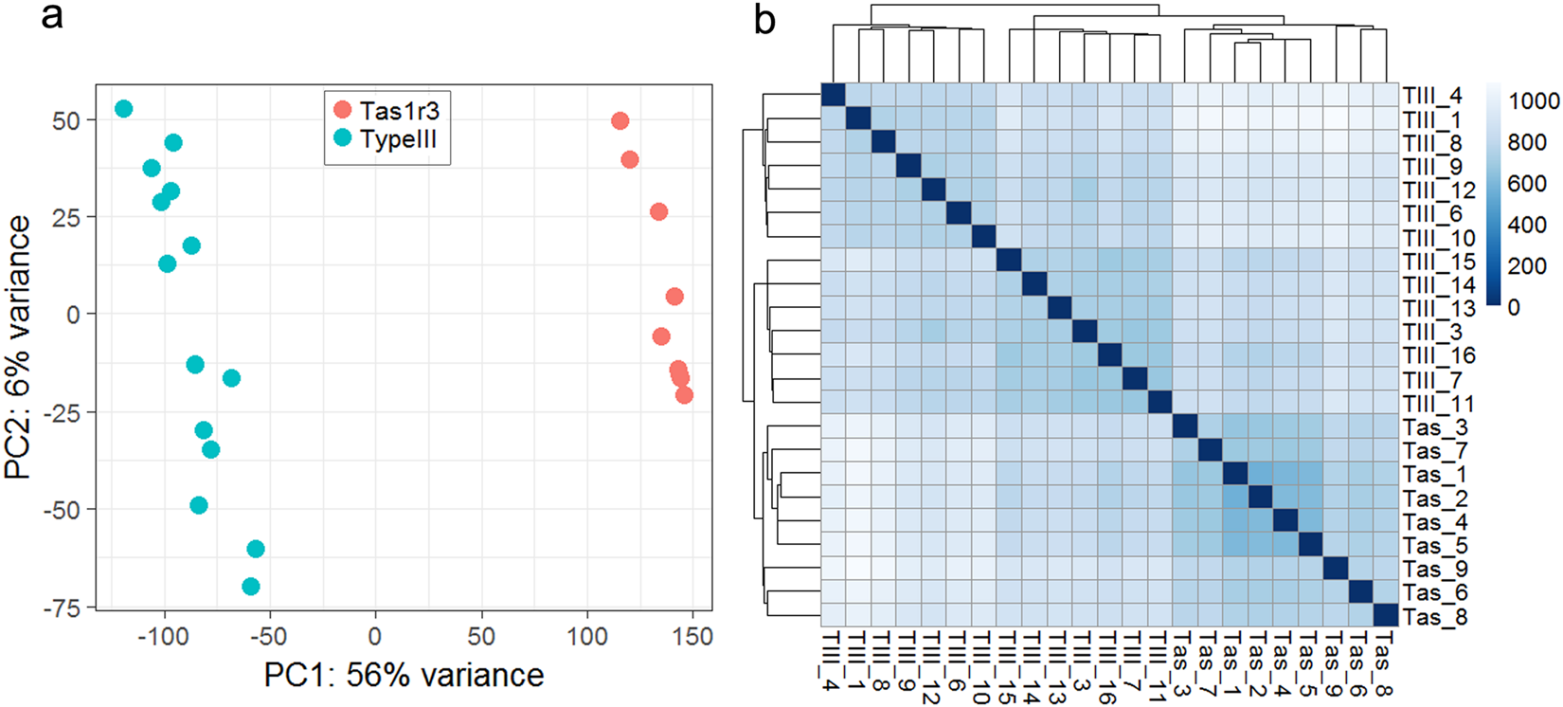
Comparisons of global gene expression among individual *Tas1r3*+ cells and type III cells. (**a**) Principal component analysis of *Tas1r3*+ cells (red) and type III cells (blue). (**b**) Hierarchical cluster analysis and heat map of Euclidean distances (shown in Table S4) among individual *Tas1r3*+ (Tas_1 through Tas_9) cells and type III cells (TIII_1 through TIII_16). Darker shades of blue indicate smaller sample distance and higher relatedness between libraries (scale bar, right). Both analyses show that transcriptomes from cognate cells are more similar to each other than to transcriptomes from cells belonging to the different group.

We next compared global gene expression within and between *Tas1r3*+ and type III cells using principal component (PC) (Fig. 1a) and hierarchical cluster (Fig. 1b, Supplementary Table S4) analyses. In both analyses, *Tas1r3*+ and type III cells formed distinct clusters based on their patterns of gene expression. Differences between *Tas1r3*+ and type III cells are mostly attributed to PC1, responsible for 56% of variance, while variation within each group of cells is mostly explained by PC2, responsible for only 6% of variance. Most of the variation in gene expression patterns is associated with differences between *Tas1r3*+ and type III cells. PC2 and the smaller clusters identified by hierarchical cluster analysis may be capturing subtypes within the *Tas1r3*+ and type III cell populations. Whether these subtypes reflect different stages of cell differentiation or represent functionally distinct groups of cells (e.g., acid- and salt-responsive type III cells) requires further investigation.

### Expression of taste cell-type-specific marker genes

To further assess the quality and specificity of our single-cell RNA-Seq data, we examined the expression of taste cell marker genes known to be differentially expressed in type II versus type III taste cells. Type II marker genes included subunits of the sweet and umami receptors (*Tas1r1*, *Tas1r2*, and *Tas1r3*) and components of their shared downstream signal transduction pathway, including *Gnat3*, *Gna14*, *Gng13*, *Plcb2*, *Trpm5* and *P2rx7* ^9, 16, 34^. Type III marker genes included *Car4*, *Gad1*, *Snap25*, *Ncam1*, *Cacna2d1*, *Chga*, *Chgb*, *Pkd2l1 and Pkd1l3* ^30, 35–39^. The type II marker genes were expressed highly in *Tas1r3*+ cells, but at very low levels in type III cells (Supplementary Fig. S3, Table 1). Conversely, type III cell marker genes showed very strong expression in type III cells, but very little if any expression in the *Tas1r3*+ cells. These results validate that the *Tas1r3*+ type II and type III cells we collected were accurately identified based on GFP fluorescence or physiological responses, respectively.

**Table 1:** RNA-Seq data for genes known to be selectively expressed by *Tas1r3*+ and type III taste cells based on evidence from the literature. “Marked cell type” = a cell type where the gene is reported to be expressed. “Log2FC” = fold change (*Tas1r3*+ vs. type III cells) in log2 scale. FDR = p values adjusted for multiple comparisons using false discovery rate (the Benjamini-Hochberg procedure implemented in the DESeq2 package).

Gene symbol	Gene name	Marked cell type	Average counts		Log2FC	FDR
			Tas1r3+	Type III		
*Plcb2*	phospholipase C, beta 2	Tas1r3+	52005	2.2	−14.4	1.10E-103
*Tas1r2*	taste receptor, type 1, member 2	Tas1r3+	3103.2	0.1	−14.8	1.10E-26
*Tas1r3*	taste receptor, type 1, member 3	Tas1r3+	6619.5	48.4	−6.7	1.58E-05
*Tas1r1*	taste receptor, type 1, member 1	Tas1r3+	1804.5	14.8	−6.4	7.14E-04
*Trpm5*	transient receptor potential cation channel, subfamily M, member 5	Tas1r3+	22979	1.5	−13.7	2.33E-62
*P2rx7*	purinergic receptor P2X, ligand-gated ion channel, 7	Tas1r3+	1624.7	30.8	−5.3	2.73E-03
*Gna14*	guanine nucleotide binding protein, alpha 14	Tas1r3+	22803	26.9	−9.1	1.37E-08
*Gng13*	guanine nucleotide binding protein, gamma 13	Tas1r3+	681.5	0.08	−12.3	8.42E-21
*Chgb*	chromogranin B	Type III	1.5	8288.3	12.1	8.38E-38
*Chga*	chromogranin A	Type III	4.7	8001.3	10.3	8.09E-21
*Ncam1*	neural cell adhesion molecule 1	Type III	30.4	16476.9	8.9	1.15E-26
*Gad1*	glutamate decarboxylase 1	Type III	2.5	33978.7	13.5	1.90E-64
*Car4*	carbonic anhydrase 4	Type III	11.3	9307	9.2	1.57E-10
*Pkd2l1*	polycystic kidney disease 2-like 1	Type III	4.5	28791.2	12.6	7.70E-158
*Pkd1l3*	polycystic kidney disease 1 like 3	Type III	23	24051.7	9.8	4.24E-30
*Snap25*	synaptosomal-associated protein 25	Type III	70.5	73176.5	9.9	1.51E-50
*Cacna2d1*	calcium channel, voltage-dependent, alpha2/delta subunit 1	Type III	11.5	26660.5	11.0	6.09E-42

Interestingly, we observed frequent co-expression of all three *Tas1r* genes in almost all the *Tas1r3*+ cells (Supplementary Table S5). Although initial reports^4, 40^ suggested that *Tas1r3* is co-expressed with either *Tas1r1* or *Tas1r2*, or sometimes with no other *Tas1r* gene, more recent work^41, 42^ using RT-PCR of single taste cells reported co-expression of all three *Tas1r* genes and physiological responses to both sweet and umami tastants. The existence of cells expressing both sweet (*Tas1r2* + *Tas1r3*) and umami (*Tas1r1* + *Tas1r3*) receptor genes is inconsistent with strict labelled line models of taste quality coding^43^ and raises the possibility of a more distributed mechanism for sweet and umami detection. It may also explain the observation in rodents that conditioned taste aversion generalizes between sweet and umami tastants^44, 45^.

### Functional significance of gene expression patterns in *Tas1r3*+ and type III cells

To identify genes that are differentially expressed between the two cell populations, we used the DESeq. 2 package. We detected 3,466 genes that are differentially expressed with absolute fold change ≥2, average expression ≥10 counts and false discovery rate (FDR) ≤0.05. Of these, 2,353 genes are up-regulated in *Tas1r3*+ cells and 1,113 genes are up-regulated in type III cells (Supplementary Table S6, Supplementary Fig. S4). To generate a functional profile of these differentially expressed genes and to elucidate the underlying cellular mechanisms and pathways differentially expressed in the *Tas1r3*+ and type III cell populations, we conducted Gene Ontology (GO) term enrichment analysis and Ingenuity Pathway Analysis (IPA).

#### Gene Ontology term enrichment analysis of differentially expressed genes

GO is a classification system where standard terms describing biological processes, cellular components and molecular function are assigned to genes and their products. The goal of the GO term enrichment analysis is to measure whether any of the GO terms occur more frequently than expected by chance (i.e., are overrepresented, or enriched) in an experimental set of genes relative to a reference or background set of genes. Typically, GO enrichment analysis identifies many redundant terms, which can make interpretation of the results difficult. The GO term summarization tool REVIGO identifies clusters of semantically similar GO terms and selects a representative GO term for each cluster, using a method conceptually similar to hierarchical clustering analyses^46^. We conducted GO term enrichment analyses comparing the differentially expressed genes from *Tas1r3*+ cells or type III cells against the background of all 43,346 annotated genes in Gencode M4. Significantly enriched GO terms in each list (p-val ≤ 10E-03) were summarized using REVIGO to generate non-redundant, representative subsets of the GO terms (Supplementary Tables S7 and S8).

**Figure 2:**
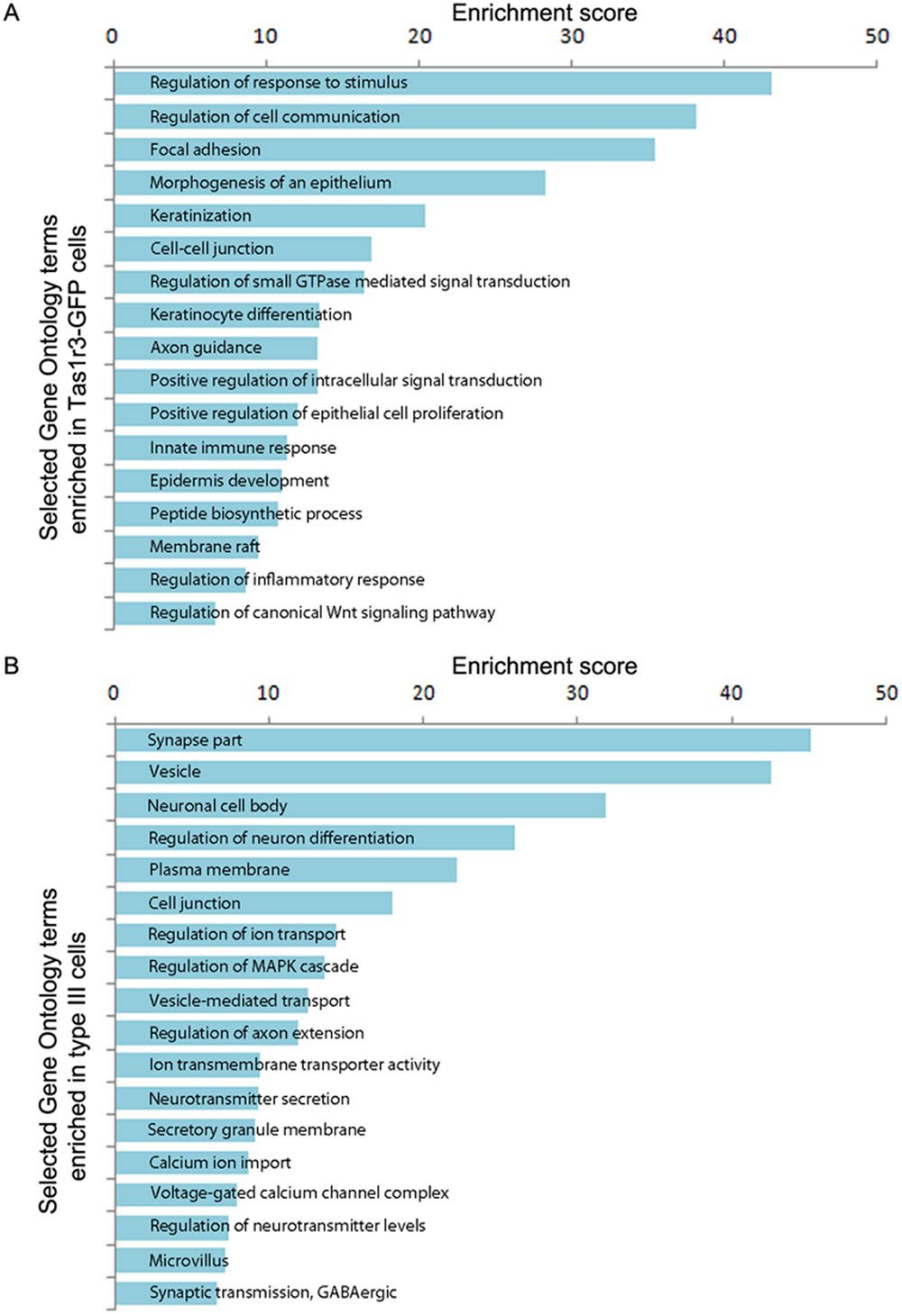
Selected Gene Ontology categories enriched in *Tas1r3*+ cells (**a**) or type III cells (**b**) relative to all annotated genes in the genome. Bar plots show some GO terms associated with previously identified biological properties of *Tas1r3*+ and type III cells identified by GO enrichment analysis.

Some prominent GO terms identified by enrichment analysis are shown in Fig. 2. *Tas1r3*+ cells are enriched for genes indicative of the epithelial origin of these cells (GO terms keratinization, keratinocyte differentiation, epidermis development, and regulation of epithelial cell proliferation). *Tas1r3*+ cells are also enriched for GO terms associated with signal transduction through membrane-associated receptors (GO terms response to stimulus, intracellular signal transduction, and membrane rafts). This corresponds to the known involvement of these cells in transduction of sweet and umami taste. Also, GO terms related to the innate immune response and inflammatory response were up-regulated in these cells, consistent with recent findings that these cells produce cytokines and other immune regulators and are affected by infection and autoimmunity^47–50^.

Type III cells are enriched for genes encoding channels (GO terms ion transport and voltage-gated calcium channels), involved in synaptic transmission (GO terms synapse part, vesicle), and indicative of the neuron-like features of these cells (GO term regulation of neuron differentiation). Previous studies have shown that type III cells have many neuron-like properties, including the ability to synthesize the neurotransmitters serotonin (5-HT), GABA, and norepinephrine; form classical synapses with gustatory nerve fibres; and fire action potentials^30, 51–54^. The enrichment of GO terms related to these neuronal functions detected in our analyses fits with the known properties of these cells. Interestingly, GO terms related to axon guidance were enriched in both cell types and could indicate an active role for these cells in orchestrating connections with gustatory nerves, as discussed below in relation to semaphorin signalling.

#### Ingenuity Pathway Analysis

We conducted IPA using a subset of 2,370 differentially expressed genes as focus genes. The major functional categories identified as up- or down-regulated by IPA are cell death and viability (Supplementary Table S9), cell movement and chemotaxis (Supplementary Table S10) and nervous system development and function (Supplementary Table S11). Among the set of functions belonging to cell death and viability, apoptosis and necrosis were up-regulated and those related to cell viability were down-regulated in type III cells compared to *Tas1r3*+ cells. (Supplementary Table S9). Similarly, in GO analysis, GO terms “regulation of cell death” and “death” were significantly overrepresented in type III cells (Supplementary Table S7). Up-regulation of apoptosis in type III cells, which were identified based on their ability to respond to KCl, could indicate that they represent a mature population of taste cells that may include many cells nearing the end of their natural life-span. It is also possible that apoptotic processes were upregulated in response to stresses associated with calcium imaging, including nutrient deprivation and exposure to ultraviolet light. In contrast, the *Tas1r3*+ population may be more diverse, consisting of cells at all stages of development and differentiation, and they were not subjected to additional stressors following isolation. Because differences in expression of cell death and viability genes may reflect differences in methods of cell collection rather than intrinsic differences between *Tas1r3*+ and type III cells, we focused further analyses in IPA only on cell migration and neuronal function pathways.

Taste cells undergo fast turnover and are continually replaced by new cells generated from the stem cell population at the base of the taste buds^55–57^. Newly generated cells have to differentiate and migrate to the appropriate position in taste buds, and the up-regulation of cellular movement pathways is significant in this context. The majority of these functions, including chemotaxis, movement of blood cells, endothelial cells, and leucocytes, were up-regulated in *Tas1r3*+ cells, while those related to movement of neurons were up-regulated in type III cells (Supplementary Table S10). Indeed, lineage tracing experiments in zebrafish and mice show that newly generated taste cells are found outside of or at the periphery of taste buds and subsequently migrate to the core of the taste buds^58, 59^. Speculating on the significance of these pathways, we note that type II cells share some properties of immune cells, while type III cells share many properties of neurons. Among the functions in IPA related to nervous system development and function (Supplementary Table S11), outgrowth of axons, migration of neurons, and long-term potentiation in hippocampus were up-regulated in type III cells, while formation of dendrites and migration of neuroglia were up-regulated in *Tas1r3*+ cells. The up-regulation of neuron-specific functions is in agreement with the neuronal properties of type III cells.

**Figure 3:**
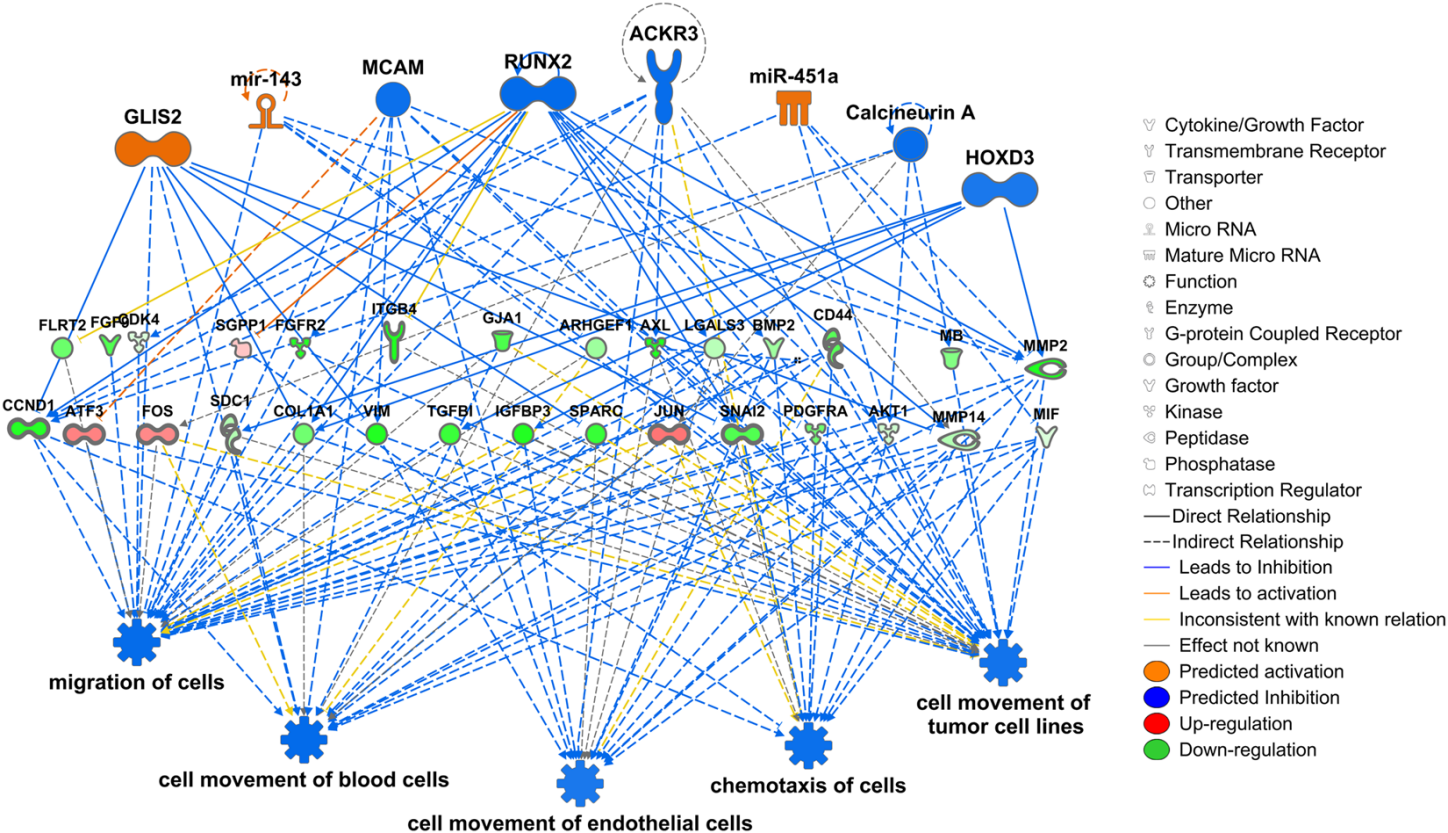
Upstream regulator effect analysis of chemotaxis and cell migration pathways using Ingenuity Pathway Analysis. Pathways that are known to influence movement of blood and tumour cells are down regulated in type III cells relative to *Tas1r3*+ cells; these functions likely influence movement of *Tas1r3*+ taste cells. The top tier in each network shows master regulators, the middle tiers show the intermediate regulators in the data set through which the master regulators exert their effect on the regulated functions, which are shown in the bottom tier. The predicted state of the regulators and affected functions are colour coded, with orange colour indicating activation and blue indicating inhibition. Up-regulated molecules in the middle tiers are colour coded red and down-regulated molecules in green, with the intensity matching the false discovery rate calculated by DESeq. 2. The shape of each molecule indicates its molecular function. The causal relationships that are consistent with Ingenuity Pathway Analysis database are represented with orange and blue lines, while the inconsistent relationships are indicated with yellow lines.

**Figure 4:**
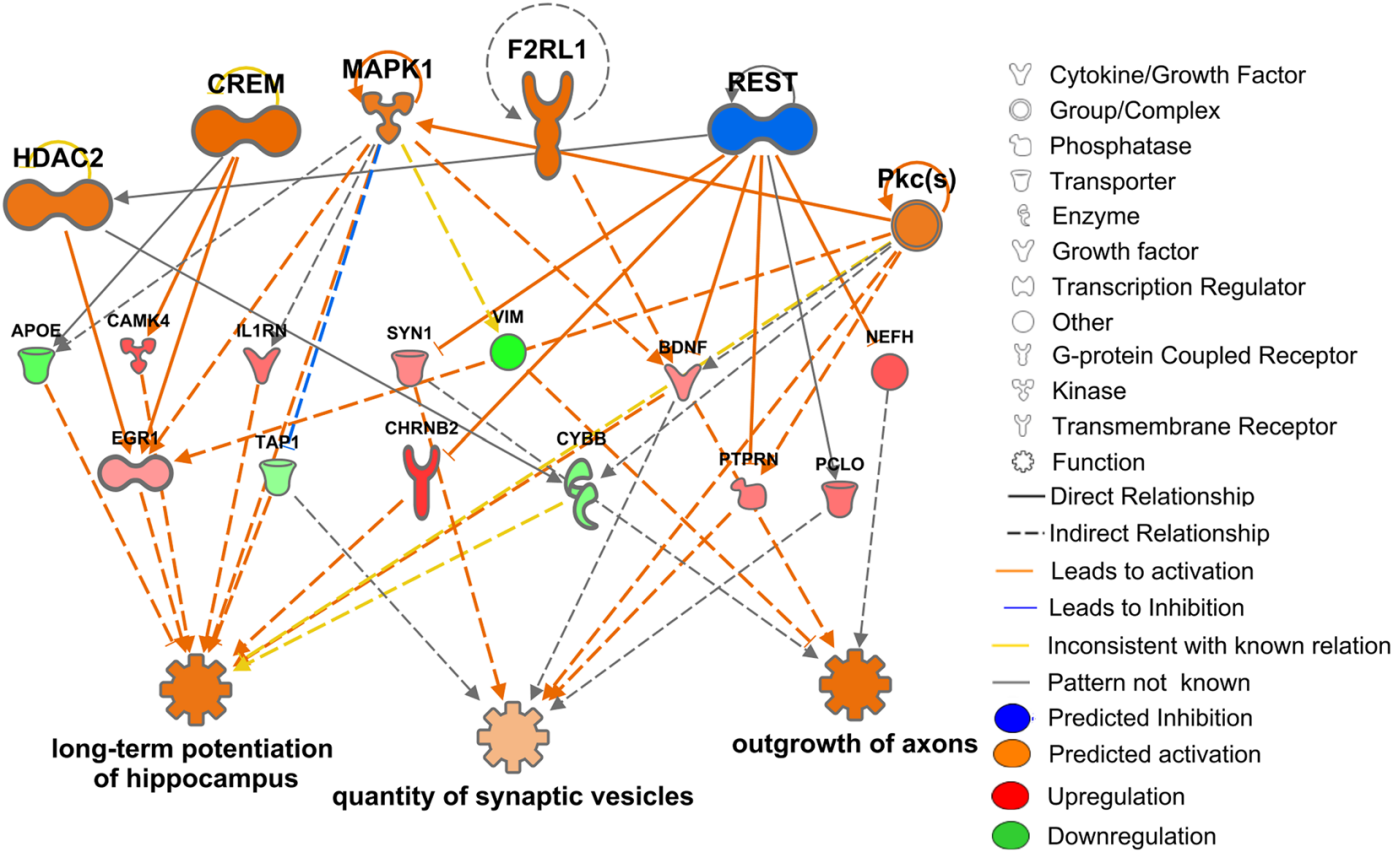
Upstream regulator effect analysis of nervous system related functions. The results show that pathways that regulate synaptic vesicle quantity, outgrowth of axons and long term potentiation pathways in hippocampus are up-regulated in type III cells. The colour coding and organization are as described in Fig. 3.

The cellular functions and diseases identified by IPA may be regulated by a variety of master regulators including transcription factors, miRNAs, and signal transduction molecules. Regulator effect analysis in IPA attempts to identify such regulators and predict their activation stage and the intermediate genes through which they exert the regulation^60^. Both GO term enrichment and IPA indicated that functions related to cell movement and the neuronal properties were upregulated in *Tas1r3*+ and/or type III cells. To explore them further, we conducted regulator effect analyses using the terms ‘cellular movement’ and ‘nervous system development and function’ as filters. The top networks generated by each analysis and their components are shown in Figs 3 and 4 and Supplementary Table S12. The master regulators for the cellular movement functions include several transcription factors, miRNAs, GPCRs, and other molecules that target 29 downstream molecules (Fig. 3). Together, they may orchestrate cell migration of *Tas1r3*+ taste cells. This analysis also identified a smaller network of genes in type III cells that may orchestrate cell migration pathways similar to those in neurons (Supplementary Table S12). With respect to neuronal functions in type III cells, 6 master regulators and 13 target genes were identified that may orchestrate synaptic vesicle formation, outgrowth of axons, and long-term potentiation of hippocampus (Fig. 4). The networks generated in these analyses may be helpful in formulating testable hypotheses on the pathways governing cell migration of taste cells and the neuron-like properties of type III cells.

### Immunohistochemical analysis of gene expression in taste cell types

GO enrichment analysis and IPA both found that functions related to cell movement and neuronal systems are prominent in *Tas1r3*+ and/or type III cells. From these broad functional categories, we selected a few genes from two specific categories known to be active in taste cells for further investigation using double-labelled immunohistochemistry: the synaptic components and the semaphorin signalling pathway, a part of the axon guidance pathway (Supplementary Tables S13–S15). We used CV papilla sections from Tas1r3-GFP mice to identify *Tas1r3*-expressing cells and sections from GAD1-GFP mice or from 5-HT-injected wild-type mice (visualized with anti-5-HT antibody) to identify type III cells.

#### Expression of synaptic component genes

**Figure 5:**
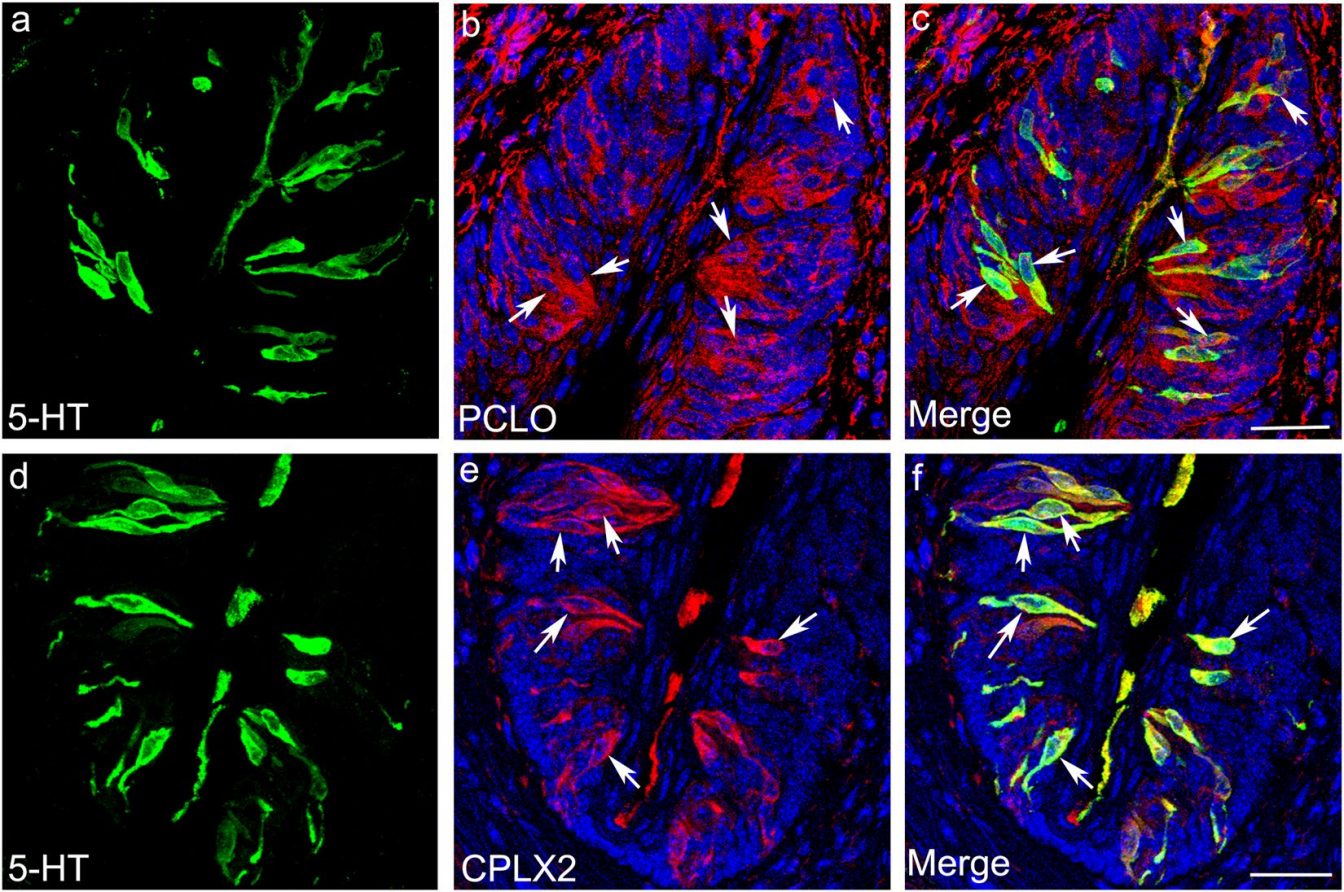
Expression of synaptic components in type III taste cells. Immunostaining was used to identify synaptic components PCLO and CPLX2 (red, **a**,**d**) and the type III marker 5-HT (green, **b,e**). Merged images (**c,f**) indicate that PCLO is expressed in almost all 5-HT expressing cells in addition to several non 5-HT expressing cells while CPLX2 is expressed exclusively in almost all 5-HT expressing cells. Arrows point to cells co-expressing 5-HT and PCLO or CPLX2. Scale bars: C = 20 µm, F = 40 µm.

The 420 synaptic component genes expressed in our data set (average count ≥20) are shown in Table S13. Of the 141 of these genes that are differentially expressed (FDR-corrected p < 0.05), 83 of them are up-regulated in type III cells and 58 in type II cells. We chose to further investigate the expression of the genes *Cplx2* and *Pclo*, which are both significantly over-expressed in type III cells (Supplementary Table S15). CPLX2 is a cytoplasmic protein that interacts with the SNARE protein complex to modulate synaptic vesicle exocytosis, while PCLO is a component of the active zone matrix and is involved in synaptic vesicle fusion and trafficking^61–64^. In agreement with previous reports and our RNA-Seq results, double-label immunohistochemistry for CPLX2 and PCLO with the type III cell marker 5-HT showed a high degree of overlap: 94.5% and 69.4% of CPLX2- and PCLO-labelled cells, respectively, were also positive for 5-HT. Within the population of 5-HT positive type III cells, 99.4% and 89.2% were immunopositive for CPLX2 and PCLO, respectively (Fig. 5, Table 2). On the other hand, only 22.5% and 48.9% of CPLX2- and PCLO-labelled cells, respectively, were immunopositive for Tas1r3-GFP, while 32 and 100% of Tas1r3-GFP cells were immunopositive for CPLX2 and PCLO, respectively (Supplementary Fig. S5, Table 2). The detection of PCLO in Tas1r3-GFP cells (Supplementary Fig. S5, Table 2) fits with our RNA-Seq results (Supplementary Table S15). While *Pclo* expression is significantly higher in type III cells, it is also expressed at lower levels in all *Tas1r3*+ cell libraries.

**Table 2:** Coexpression of the synaptic vesicle components (CPLX2 and PCLO) and the semaphorin signalling components (SEMA4A, PLEXINB1, CRMP2 and FES) with (A) TAS1R3-GFP, (B) 5HT and (C) GAD1-GFP in circumvallate papillae taste cells of mice.

**A: Number of taste cells expressing TAS1R3-GFP and target proteins**				
**Target protein**	**Target protein + cells**	**TAS1R3-GFP + cells**	**Coexpressed/Target protein**	**Coexpressed/TAS1R3-GFP**
CPLX2	80	56	18/80(22.5%)	18/56 (32%)
PCLO	98	48	48/98 (48.9%)	48/48 (100%)
SEMA4A	98	65	55/98 (56.1%)	55/65 (84.6%)
PLEXINB1	106	54	52/106 (49%)	52/54(96.3%)
CRMP2	92	58	49/92 (53.2%)	49/58 (84.5%)
FES	219	138	123/219 (56.1%)	123/138 (89.1%)
**B: Number of taste cells expressing 5HT and target proteins**				
**Target protein**	**Target protein + cells**	**5HT + cells**	**Coexpressed/ Target protein**	**Coexpressed/5HT**
CPLX2	183	174	173/183 (94.5%)	173/174 (99.4%)
PCLO	108	84	75/108 (69.4%)	75/84 (89.2%)
FES	130	39	16/130 (12.3%)	16/39 (41%)
SEMA4A	110	50	24/110 (21.8%)	24/50 (48%)
**C: Number of taste cells expressing GAD1-GFP and target proteins**				
**Target protein**	**Target protein + cells**	**GAD1-GFP + cells**	**Coexpressed/ Target protein**	**Coexpressed/ GAD1-GFP**
PLEXINB1	175	111	40/175 (22.8%)	40/111 (36.0%)
CRMP2	107	88	56/107 (52.3)	56/88 (63.6%)

#### Expression of semaphorin signalling pathway genes

**Figure 6:**
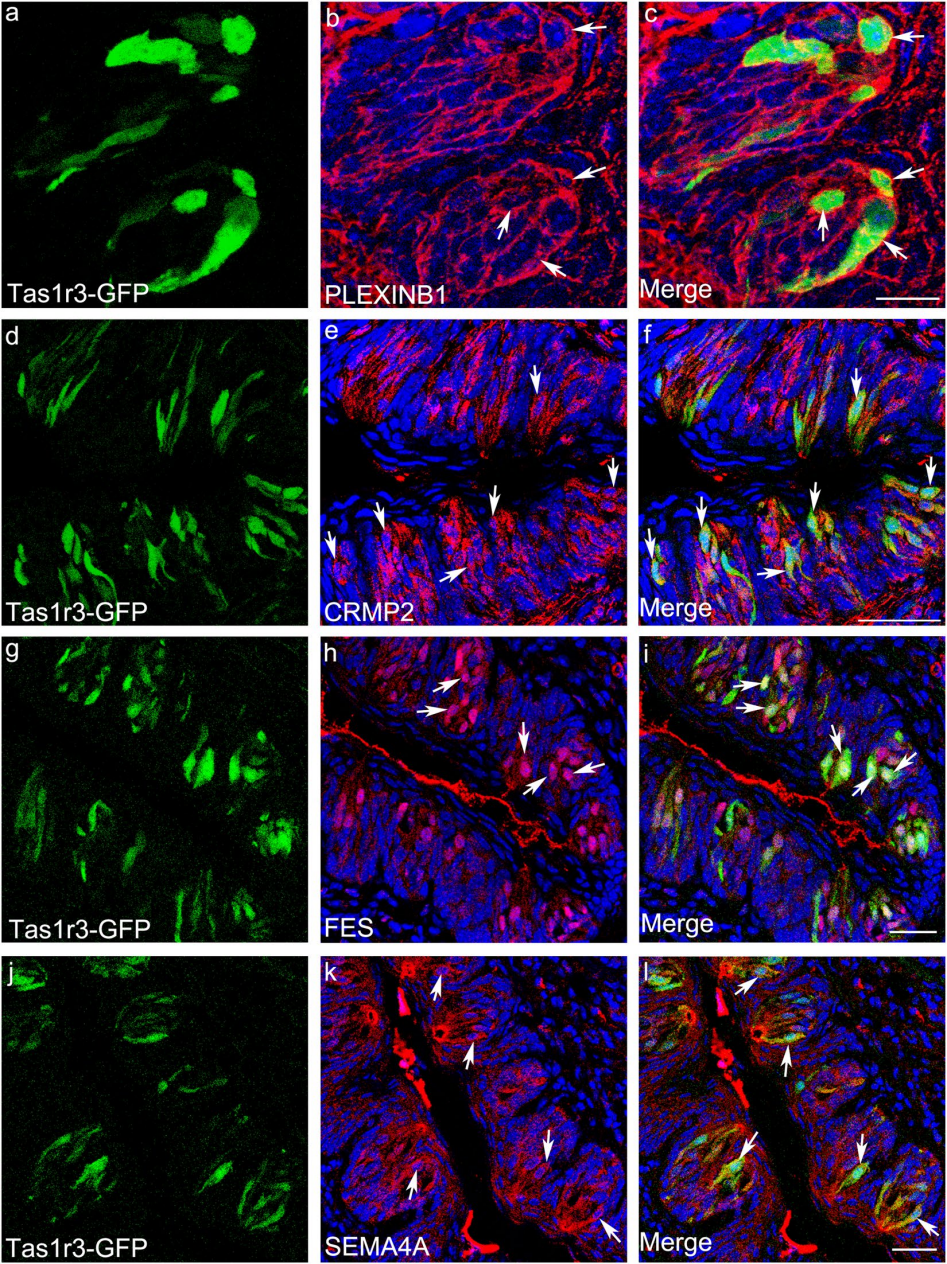
Expression of semaphorin pathway genes in *Tas1r3*+ taste cells. Immunostaining identifies the semaphorin pathway components PLEXINB1, CRMP2, FES and SEMA4A (red, **a,d,g,j**). Intrinsic fluorescence of Tas1r3-GFP identifies *Tas1r3*+ taste cells (green, **b,e,h,k**). Merged images (**c,f,i,l**) indicate that most *Tas1r3*+ cells express the semaphorin pathway components examined, and that they are expressed in other taste cells also. Arrows point to cells co-expressing Tas1r3-GFP and PLEXINB1, CRMP2, FES or SEMA4A. Scale bars: c = 16 µm, f, i, l = 40 µm.

We identified components of the semaphorin signalling pathway that are abundantly expressed in taste cells (Supplementary Fig. S6, Supplementary Table S14). We chose the genes *Sema4a, PlexinB1*, *Fes*, and *Crmp2* (*Dpysl2*) for further validation. SEMA4A is a secreted semaphorin molecule, and PLEXINB1 is its cognate receptor (Supplementary Fig. S6)^65, 66^. FES is a kinase that phosphorylates CRMP2, itself a kinase involved in remodelling the microtubules in the cytoskeleton^67, 68^. Both FES and CRMP2 are downstream components of the SEMA3 signalling cascade (Supplementary Fig. S6). In our RNA-Seq data set, the genes *Sema4a* and *Fes* are expressed at significantly higher levels in *Tas1r3*+ cells than in type III cells. Consistent with this, immunohistochemical studies showed that 56.1% of SEMA4A- or FES-expressing cells express Tas1r3-GFP, and 84.6 and 89% of *Tas1r3*-expressing cells express SEMA4A and FES, respectively (Fig. 6, Table 2). Also in agreement with the RNA-Seq results, SEMA4A and FES immunoreactivity was less prevalent in type III cells. Only 21.8% of SEMA4A expressing were 5-HT positive and only 48% of 5-HT expressing cells were SEMA4A positive (Supplementary Fig. S7, Table 2). Similarly, only 12.3% of FES-expressing cells were 5-HT positive, and only 41% of 5-HT-expressing cells were positive for FES (Supplementary Fig. S7, Table 2). Interestingly, FES is localized to the nucleus in taste cells (Supplementary Fig. S7d–f and Fig. 6i–l). Nuclear localization has deen reported for FES in other cell types, and its sub-cellular localization is thought to vary depending on the cell type and/or stage of cell cycle^69^. Significant expression of *PlexinB1* and *Crmp2* was detected in the RNA-Seq libraries of both *Tas1r3*+ and type III cells. Immunohistochemical studies showed similar results, though PLEXINB1 immunoreactivity was less common in type III cells than the RNA-Seq data would predict. Among CRMP2- and PLEXINB1-expressing cells, 53 and 49%, respectively, express Tas1r3-GFP, while 84.5 and 96% of Tas1r3-GFP-positive cells express CRMP2 and PLEXINB1 respectively (Fig. 6). In addition, 52.3% and 22.8% of CRMP2- and PLEXINB1-expressing cells, respectively, expressed the type III marker GAD1-GFP, while 63.6 and 36% of GAD1-GFP cells express CRMP2 and PLEXINB1, respectively (Supplementary Fig. S7, Table 2). In control experiments, CRMP2 immunoreactivity was blocked by preincubation with the peptide used to raise the antibody (Supplementary Fig. S8).

## Discussion

In this study we have generated single-cell RNA-Seq libraries from *Tas1r3*-expressing type II taste cells and physiologically-identified type III taste cells. Single-cell RNA-Seq data is intrinsically noisy due to a combination of intrinsic biological variability and technical variation^32, 70^. Robust differential expression analysis using RNA-Seq data derived from bulk tissue samples requires only two to three replicates per condition; for single-cell RNA-Seq data more replicates are required^32, 70^. Using single-cell RNA-Seq data from 9 *Tas1r3*+ and 14 type III cells, we were able to conduct a robust differential expression analysis that accurately captured the known expression patterns of taste cell marker genes. Further, we derived many biologically meaningful insights using GO enrichment and IPA. For example, GO analysis (Fig. 2, Supplementary Tables S7 and S8) and IPA (Fig. 4) of our RNA-Seq libraries support the importance of axon guidance pathways in taste tissue.

To further validate our single-cell RNA-Seq data and demonstrate one of the practical applications of mining transcriptome data, we identified strongly expressed genes associated with synapses or the semaphorin signalling pathway that had seen little to no prior study in taste tissue. Using immunohistochemistry, we confirmed the expression of these genes in taste cells, the details of which are discussed below.

The presence of synaptic vesicles and active zones is one of the defining characteristics of type III (presynaptic) taste cells, and numerous synaptic component genes have been investigated in taste tissue^36, 71–74^. Expression of *Cplx2* has been reported in type III taste cells^61^, but there were no previous reports of *Pclo* expression. The formation of precise connections between neurons and between neuronal and non-neuronal cells is orchestrated by axon guidance pathways. To date, most studies of axon guidance pathways in the taste system were conducted in embryos^75–78^. Adult taste cells live only 3–24 days, and new cells are continually generated from stem cells^79^; thus, axon guidance pathways must function throughout the lifetime in taste buds. Furthermore, taste neurons can find their way to the taste papillae after experimental taste nerve transection in adults^80, 81^, which would require axon guidance pathways. Among axon guidance pathways, the Semaphorin pathway is well studied in embryonic taste placodes, where *Sema3a* and *Sema3f* act to repel or suppress geniculate and trigeminal axons and presumably fine-tune the timing with which these axons penetrate the taste papillae^75, 76^. Overall, there was good agreement between our single-cell RNA-Seq data and immunohistochemistry. For example, both immunohistochemistry and RNA-Seq data agree that expression of CPLX2 and PCLO is high in type III cells and low in Tas1r3-GFP cells. However, some data were less consistent. For example, FES expression was not detected in RNA-Seq libraries from type III cells, but ~41% of type III cells were immunopositive for FES. All type III cell RNA-Seq libraries showed strong expression of CRMP2, while only 63.6% of GAD1-GFP type III cells were immunopositive for CRMP2.

Numerous factors could have contributed to these inconsistencies. First, we only sequenced a relatively small number of cells from each population and may not have captured the full range of natural variability within the *Tas1r3*+ and type III cell subpopulations. If this is the case, newer approaches using more replicates may resolve this discrepancy. Second, single-cell mRNA amplification is prone to drop-out events and other distortions caused by inefficiency or outright failure of reverse transcription and *in vitro* transcription of some genes^32^. Third, the type III cells used for RNA-Seq were isolated based on calcium responses to KCl stimulation and may represent different subpopulation(s) of type III cells than the 5-HT containing or GAD1-expressing type III cell subpopulations used in immunohistochemical analyses. Fourth, the aRNA method produces 3′ biased sequencing data, and false negatives will be obtained in cases of genes with un-annotated 3′ ends^70^. Generating taste-bud-specific gene annotations *de novo* from bulk taste RNA-Seq data will be very helpful in analysing such data. Fifth, the calcium imaging protocol, which involves incubation in nutrient-free Tyrode’s solution and exposure to ultraviolet light and KCl, subjects the cells to metabolic and other stresses that could have affected their transcription profile. This could also help explain the expression of fewer genes and the up-regulation of cell death pathways in these cells (Supplementary Tables S1, S2, & S9). However, calcium imaging did not seem to affect the expression of type-III-specific internal control genes (Supplementary Fig. S3, Table 1). To our knowledge, this is the first report of RNA-Seq data from single cells physiologically identified by calcium imaging, and our methodology can be applied to similar studies in other systems.

The single-cell RNA-Seq methodology we describe here can be applied to other subpopulations of taste bud cells (e.g., type I cells, bitter-responsive type II cells) and could eventually result in a complete model of the genes expressed by all the individual cell types that compose this important sensory organ. Such analyses should provide key insights into the receptors and signalling pathways that underlie taste perception at the periphery. Furthermore, if a baseline of gene expression under standard conditions is assembled, it could be used as a point of reference to analyse how different conditions (e.g., diet, health, circadian rhythm, blood glucose level) alter gene expression in taste cells. Our approach also provides a technical foundation that will enable future studies in other tissues that are similarly challenging to study.

## Materials and Methods

### Animals

All animal experimental procedures were approved by the Monell Animal Care and Use Committee, following National Institute of Health guidelines. Two-three month old male C57BL/6Ncrl mice (Strain code: 027, Charles River Laboratories, Wilmington, MA) Tas1r3-GFP transgenic mice^82^ and Gad1-GFP transgenic mice^83^ were used for this study.

### Isolated taste cell preparation

Mice were euthanized by exposure to CO_2_ followed by cervical dislocation. The tongue was quickly excised and washed in chilled Tyrode’s solution, and a protease mixture consisting of 1 mg/mL collagenase A (Worthington Biochemical Corp., Lakewood, NJ), 2 mg/mL dispase II (Sigma), and 1 mg/mL trypsin inhibitor, soybean purified (Worthington Biochemical Corp.) dissolved in calcium-free Tyrode’s solution was injected under the lingual epithelium. After a 12 min incubation at 37 °C, the lingual epithelium was peeled, washed, and subsequently incubated in Ca^2+^-free Tyrode’s for 30 min at room temperature. Ca^2+^-free Tyrode’s solution was replaced immediately before cell collection with DMEM for collecting GFP-labeled cells or with normal Tyrode’s solution for calcium imaging. Isolated taste cells from the circumvallate (CV) papillae were collected using pulled glass pipettes (DMZ-Universal Puller; Zeitz Instruments, Martinsried, Germany) and expelled onto coverslips (10 mm diameter; Ted Pella, Inc., Redding, CA) coated with Cell-Tak (BD Biosciences, San Jose, CA). Tas1r3-GFP cells were collected immediately after dissociation. To functionally identify type III cells, the isolated cells were allowed at least 60 min to settle onto the coverslips prior to functional imaging.

### Ca^2+^ imaging reagents and solutions

Tyrode’s solution was composed of the following (in mM): 140 NaCl, 5 KCl, 4 CaCl_2_, 1 MgCl_2_, 10 HEPES, 10 glucose, 1 Na-pyruvate, pH 7.4. Ca^2+^-free Tyrode’s solution, used during isolated taste cell preparation, was composed of the following (in mM): 140 NaCl, 5 KCl, 10 HEPES, 10 glucose, 1 Na-pyruvate, 2 EGTA, pH 7.4. The 50 mM KCl stimulus was dissolved in Tyrode’s solution with an equimolar substitution of KCl for NaCl. The pH of all solutions was adjusted to 7.4 using NaOH or HCl. Reagents were obtained from Thermo Fisher Scientific (Waltham, MA) unless otherwise noted.

### Calcium imaging

Isolated taste cells were transferred to an imaging chamber (RC-26; Warner Instruments Inc., Hamden, CT) and loaded with fura-2 AM (6.5 µM; Invitrogen, Carlsbad, CA) containing the dispersing agent Pluronic F-127 (Molecular Probes, Invitrogen) for 30 min at 37 °C. Calcium imaging was conducted using an Olympus BX51-WI upright fixed-stage microscope (Olympus America, Melville, NY), MetaFluor for Olympus control software, a Hamamatsu C9100 digital camera (Hamamatsu Photonics K.K., Hamamatsu City, Japan), and Sutter Instruments Lambda 10-2 optical control system (Sutter Instruments Co., Novato, CA). Cells were kept under constant perfusion with Tyrode’s solution between stimulus presentations. In preparation for stimulus presentation, perfusion influx was interrupted and fluid levels in the recording chamber were allowed to decrease to ~20% of maximum volume; 50 mM KCl stimulus was then gently pipetted directly into the imaging chamber, filling it to 100% volume and maintaining it at this level for 4 s, after which bath solution perfusion was resumed. Fura-2 calcium signals were measured at a capture rate of 2 s during stimulus presentations, with excitation wavelengths of 340 nm and 380 nm and emission measured at 510 nm. To reduce cellular exposure to ultraviolet light and photo bleaching of the fura-2 fluorophore, the sampling rate was reduced (4–8 s) during the prolonged and roughly linear recovery phase of calcium responses, and further reduced (30–60 s) during baseline periods between stimulus presentations. Calcium levels were estimated using the ratio of fluorescent intensities at 340/380 nm and are reported in arbitrary units (AU). Data analysis was conducted using custom scripts written in MatLab (MathWorks Inc., Natick, MA). An average baseline signal was calculated from the 120 s prior to stimulus presentation. A significant response to 50 mM KCl was defined as an increase in the F_340_/F_380_ signal that remained >10 SD above baseline levels for 10 s consecutively.

### Cell Collection and aRNA amplification

Individual cells were collected by aspiration using a micro capillary tube attached to a Leitz micromanipulator (Leica Microsystems, Buffalo Grove, IL) and expelled into PCR tubes containing 2 μL QuickExtract RNA Extraction Solution (catalog no. QER09015; Epicentre, Madison, WI). Two rounds of aRNA amplification from this cell lysate were carried out using the TargetAmp 2-Round aRNA Amplification Kit 2.0 (catalog no. TAU2R51224; Epicentre) per manufacturer instructions. First- and second-round aRNA was purified using the RNA Clean & Concentrator-5 kit (catalog no. R1015, Zymo Research, Irvine, CA). Second-round aRNA was quantified using Nanodrop 1000 spectrophotometer (Nanodrop products, Wilmington, DE).

### RNA-Seq library preparation and sequencing

Sequencing libraries were prepared from 1 μg aRNA using the NEBNext mRNA LibraryPrep Master Mix Set for Illumina (catalog no. E6110; New England Biolabs, Ipswich, MA). aRNA was fragmented for 2 min instead of the kit-recommended 5 min to account for its shorter length. The rest of the protocol was per manufacturer instructions. Libraries were prepared with indices 4, 6, or 12 from the NEBNext Multiplex Oligos for Illumina (Index Primers Set 1, catalog no. E7335; New England Biolabs). Libraries were quantified using the KAPA Universal Library Quantification Kit (catalog no. KK4824; Kapa Biosystems, Wilmington, MA); three libraries with indices 4, 6, and 12 were multiplexed in equimolar concentration, and 100-bp paired-end sequencing was done on a HiSeq. 2000 sequencer (Illumina, San Diego, CA) using standard Illumina sequencing protocols.

### Genomic alignment of RNA-Seq reads

Raw sequences in fastq format were aligned to the mouse reference genome (version GRCm38.p3) using the STAR program with default settings that include soft clipping, and Gencode M4.gtf as the splice junction annotation file^84^. STAR output files in BAM format were sorted and indexed with SAMtools^85^. To reduce alignment of repetitive reads, a multi-read correction was used allowing a maximum of 10 alignments per read.

### Gene expression calculation and quality control of RNA-Seq results

Reads mapping to the exons of genes were quantified using featureCounts with the *Mus musculus* Gencode M4 gene annotations (https://www.gencodegenes.org/mouse_releases/4.html) as reference^86^. Because of the 3′ bias introduced by oligo(dT) primed aRNA amplification, information on alternate splicing and transcription initiation is poorly represented in our data, so we conducted only a gene-level analysis. Normalization and differential expression analysis (type III/*Tas1r3*+) were done using the DESeq2 package in R/Bioconductor^33, 87^. Genes that are not expressed in any of the libraries (0 counts in all libraries) were excluded from further analysis. By default, DESeq2 excludes from FDR calculation genes with very low counts that are not likely to produce statistically significant differences using the independent filtering option. The FDR for these genes are shown as “NA”. A variance stabilizing transformation was applied to read counts used for principal component analysis, Euclidean distance calculation, and heat map generation (http://www.bioconductor.org/help/workflows/rnaseqGene). Data from the 500 genes with the highest row variance were used for principal component analysis. Euclidean distances for hierarchical cluster analysis were calculated using values from all genes expressed in our dataset with the “dist” function in R. Gene body coverage was generated using geneBody_coverage.py module in RseQC using the expression profile of the thousand most highly expressed genes across all libraries. RSeQC divides the gene body into one hundred quantiles and calculates coverage across each quantile from input BAM files. Coverage was normalized to the quantile with maximum coverage, which is designated as 100%^88^. Students T test (one tailed, unequal variance) comparing RNA-Seq data from *Tas1r3*+ and type III cells were done in Excel.

### Gene ontology term enrichment analysis

The lists of differentially expressed genes in type II (n = 2,353) and type III (n = 1,113) cells with average count ≥ 10, absolute fold change ≥ 2 and FDR ≤ 0.05 were extracted from Table S6. GO enrichment analyses were done against a background population of all genes in Gencode M4 using Partek genomics suite (PGS, Partek Inc, St.Louis, MO). PGS ranks GO terms by enrichment score, which is calculated using a chi-square test comparing the proportion of genes mapping to a term in the experimental gene list compared to the background gene list. GO terms with *p* < 10E-03 were used as input for summarization using REVIGO^46^.

### Ingenuity pathway analysis

DESeq2 results were uploaded to IPA (https://www.qiagenbioinformatics.com/products/ingenuity-pathway-analysis/). To speed up IPA analysis, we used a smaller subset of 2,370 differentially expressed genes with average count ≥ 50, absolute fold change ≥ 2 and FDR ≤ 0.05 as focus genes. In results of DESeq. 2 analysis (used as input for IPA), type III cells were used as the numerator and *Tas1r3*+ cells were used as the denominator. Therefore, in the IPA output “activated” or “upregulated” indicates higher expression in Type III cells compared with *Tas1r3*+ cells, and “inhibited” or “downregulated” indicates higher expression in *Tas1r3*+ cells compared with type III cells. Regulator effect networks were generated using the terms “cellular movement” and “nervous system development and function” as filters in the regulator effects module in IPA. My Pathways in IPA was used to create images for the pathways with the highest IPA consistency score in each analysis.

### Immunohistochemistry

2–4 month old male or female mice were euthanized by cervical dislocation. For 5-HT detection, the C57BL/6 mice were injected with 80 mg/kg 5-hydroxy-L-tryptophan (H9772, Sigma) 1 h before euthanasia. The CV-papilla-containing portions of the tongue were dissected and briefly rinsed in ice-cold PBS. Tissues were fixed for 1 h at 4 °C in 4% paraformaldehyde/1 × PBS and cryoprotected in 20% sucrose/1 × PBS overnight at 4 °C before embedding in OCT. Sections (8–10 μm thickness) were prepared using a CM3050S cryostat (Leica Microsystems) and applied on precoated microscope slides (Superfrost plus; Fisher Scientific). Sections were dried at 40 °C for 20 min and stored at −80 °C for further use. Frozen sections were rehydrated with PBS before staining. Nonspecific binding was blocked with Superblock (Thermo Fisher Scientific, Catalog #37518) at room temperature for 1–2 h. Sections were incubated with rabbit polyconal antibodies (Abcam, Cambridge, MA) against mouse CRMP2 (1:200; catalog no. ab36201, RRID: AB_731750), FES (1:200; catalog no. ab153841), SEMA4A (1:100; catalog no. ab70178, RRID:AB_1270611), PLEXINB1 (1:200; catalog no. ab90087, RRID:AB_2050196), CPLX2 (1:200; catalog no. ab101857, RRID:AB_10710874), or PCLO (1:150; catalog no. ab20664 RRID:AB_777267) overnight at 4 °C in a humidified chamber. For peptide blocking experiments, CRMP2 antibody was preincubated with tenfold excess of the immunogenic peptide (Abcam, catalog #ab196858) before incubation with tissue sections. Specific taste cell type markers were used to label type III cells in C57BL/6 mice (goat anti-serotonin [5-HT], 1:500; ab66047; Abcam, Cambridge, MA). After three 15-min washes with PBST, slides were incubated for 1 h at room temperature with Alexa647 donkey anti-rabbit fluorescent secondary antibody (1:750; ThermoFisher Scientific, Catalog#: A-31573) in blocking buffer for immunofluorescence along with DAPI (1: 1,000; Invitrogen) to label cell nuclei. In case of double labeling with 5-HT, alexa 488 anti-goat fluorescent antibody (1:700, Thermo Fisher Scientific, Catalog #: A-11055) was also added.

### Imaging

Fluorescent images were captured with the TCS SP2 Spectral Confocal Microscope (Leica Microsystems, Inc., Wetzlar, Germany). Scanware software (Leica Microsystems, Inc.) was used to acquire z-series stacks at a step size of 0.25–0.35 μm. Images were scanned using a 512 × 512-pixel format; scan lines were averaged twice, and frames were scanned three times. Digital images were cropped and arranged using Photoshop CS (Adobe Systems, Inc., San Jose, CA). Fluorescence images within a figure were adjusted for brightness and contrast for background standardization.

#### Counting immunolabeled taste cells

Quantitative measurements were conducted to determine the percentage of singly- and doubly-labeled Tas1r3-GFP+ and type III taste cells that coexpressed each protein of interest To quantify coexpression, 4–5 sections from two mice were counted for each taste cell marker. To avoid counting the same cells more than once, sections separated from each other by at least 40 µm were chosen. Nuclear staining with DAPI was used to help distinguish individual taste cells. Only cells with entire cell bodies and nuclei visible were used for counting. Cells expressing the protein of interest and taste marker-labeled taste cells were counted in respective single-channel images, then the doubly-positive cells were counted using overlaid images.

### Sequence data

All sequence data used for this study have been deposited in NCBI’s short read archive (SRA) with the accession number SRP094673.

## Electronic supplementary material

Supplementary Information

Supplementary tables
