# Supplementary material for: Whole transcriptome profiling of taste bud cells
Source: Sci Rep. 2017 Aug 8;7:7595. doi: 10.1038/s41598-017-07746-z (PMC5548921; doi:10.1038/s41598-017-07746-z)
Supplement: Supplementary file 1 — Supplementary Information [file 41598_2017_7746_MOESM1_ESM.pdf]

Supplementary information for:

Whole transcriptome profiling of taste bud cells

Sunil K. Sukumaran[1¶], Brian C. Lewandowski[1¶], Yumei Qin[1,2], Ramana Kotha[1], Alexander A. Bachmanov[1] and Robert F. Margolskee[1*]

[1]Monell Chemical Senses Center, 3500 Market Street, Philadelphia PA USA 19104

[2]Present address: College of Food & Biology Engineering, Zhejiang Gongshang University, Hangzhou 310018, P. R. China.

[*]To whom correspondence should be addressed: rmargolskee@monell.org

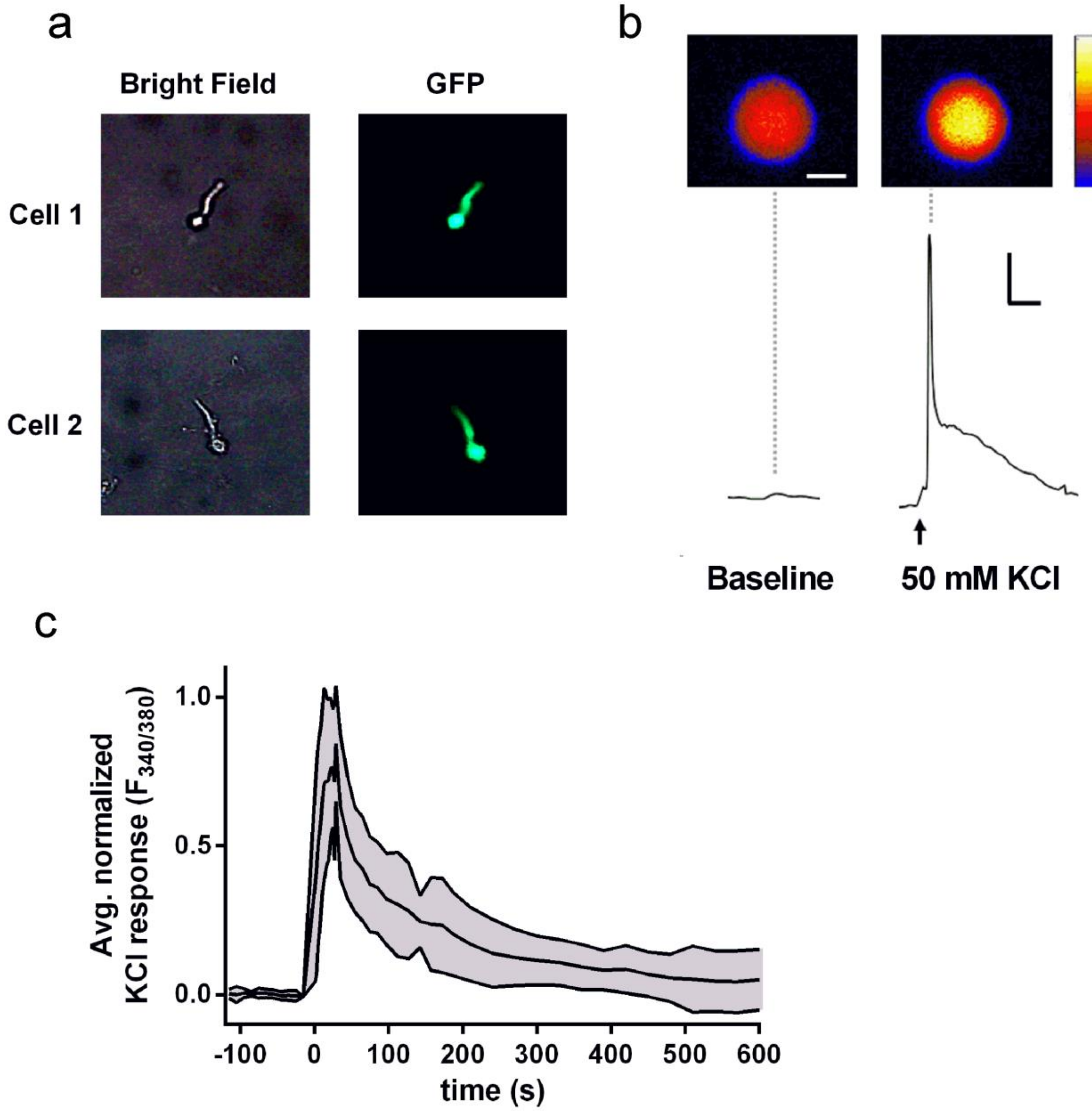

Supplementary Figure S1. Identification of *Tas1r3*-expressing (*Tas1r3*+) type II cells and KCl-responsive type III cells isolated from mouse circumvallate papillae. (a) Exemplar of isolated *Tas1r3*+ type II taste cells identified by GFP fluorescence. Images show the cells under bright field (left) and GFP fluorescence (green, right). (b) Representative data from an isolated type III taste cell exhibiting a robust 50 mM KCl depolarization induced calcium response (Fura2). Top, pseudo-colour images of calcium levels ($F_{340nm/380nm}$) in the cell during rest (Baseline, left) and

following 50 mM KCl stimulation (right). Bottom, traces of calcium levels ($F_{340nm/380nm}$) during baseline and following application of the KCl stimulus. The vertical dotted lines indicate the time-point in each trace depicted in the pseudo-coloured images. Following isolation from the taste bud, taste cells gradually lose their polarized form and become rounded. The cells in (a) were collected immediately following isolation and thus retain their polarized shape; the cell in (b) was imaged approximately 2 hours after isolation and has lost its polarized shape. (c) Average normalized calcium responses to 50 mM KCl from the 14 physiologically isolated type III taste cells whose transcriptome libraries met quality control standards (see Results). Data from individual traces was aligned to KCl stimulus onset (time 0). The thick black line shows the average response. The shaded area above and below the curve represents +/- SD. Individual KCl response traces were normalized by subtracting the average baseline calcium signal (calculated from the 120 s prior to KCl stimulation) and then dividing by the peak value of the KCl-induced calcium response. Much of the variability captured by the shaded +/- SD is explained by two factors: (1) for reasons detailed in Methods, stimuli were hand-pipetted into the recording chamber and then a time-stamp was manually added to the recording file. This process introduced an estimated 1-5 s of human error in the time stamps representing KCl stimulus onset. This likely accounts for some of the high variability near the peak of the average KCl response curve. (2) There was considerable variability in the time cells took to return to baseline calcium levels following a KCl-induced calcium response. This can be seen in the persistently high variability at later points in the average KCl response curve.

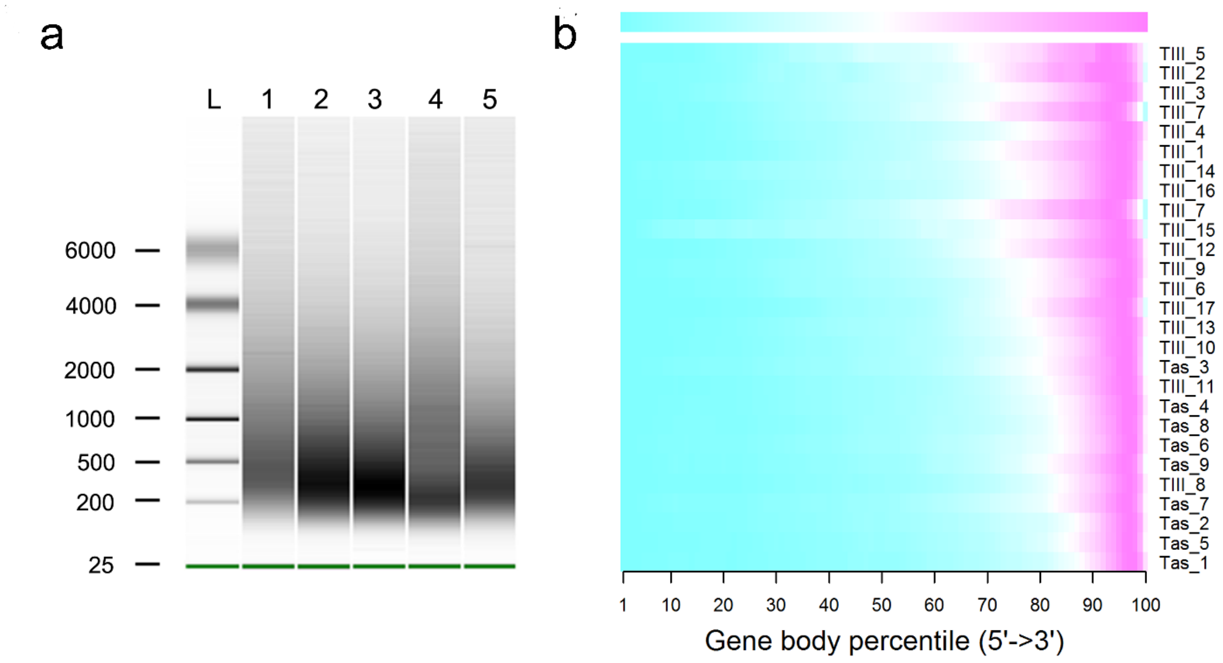

Supplementary Figure S2: Size distribution (a) and 3’ end bias (b) of aRNA samples. (a) Bioanalyzer image of five representative aRNA samples (1-5) shown along with RNA ladder (L). The bulk of the aRNA range in size from 200 - 700 nt. (b) Heat map showing coverage of RNA-Seq reads across the gene body, from the 5’ (left) to 3’ (right) end. The regions with the higher coverage are indicated with darker shades of pink and those with the lower coverage in blue (scale bar, top). As expected for aRNA samples, most of the RNA-Seq reads map to the 3’ end of genes.

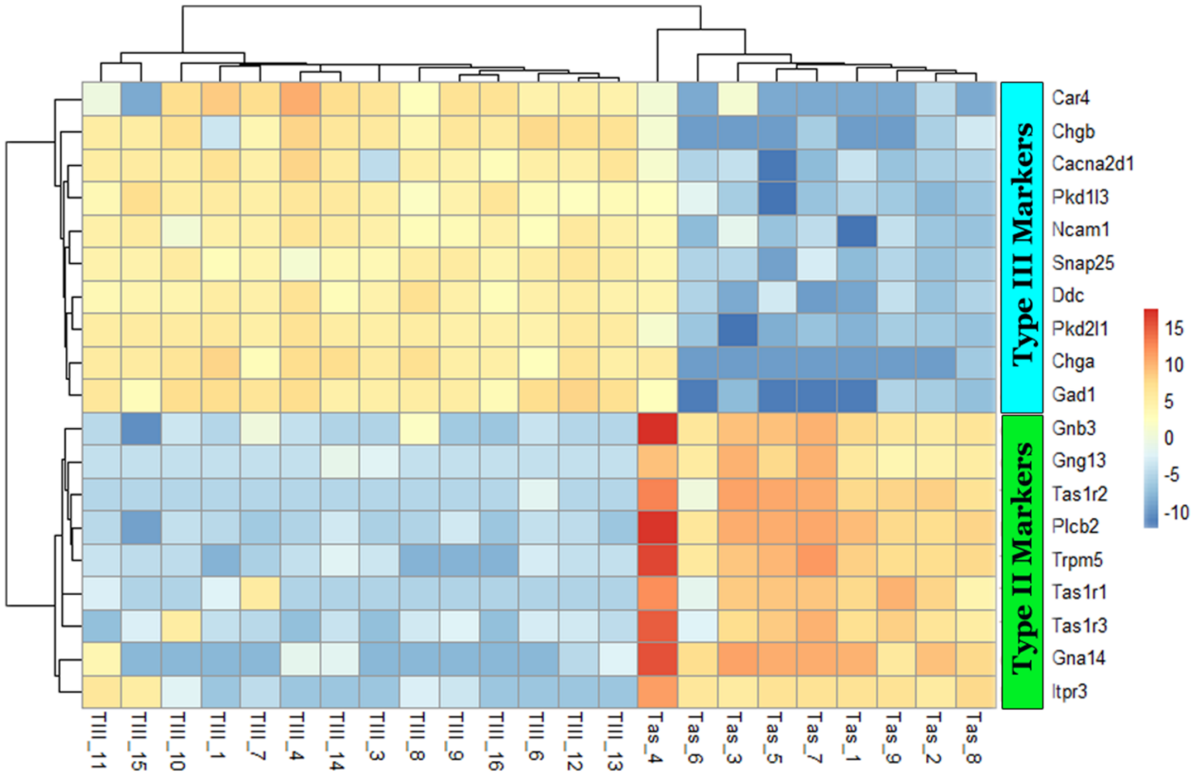

Supplementary Figure S3: Heat map and cluster analysis of expression of known taste cell marker genes in single cell RNA-Seq libraries. The variance stabilized expression value of marker genes in individual libraries was subtracted from the mean for that gene across all libraries before heat map generation and clustering. Warmer colors indicate higher expression levels (scale bar, right). Type III cell marker genes are most highly expressed in type III cells and type II cell marker genes are most highly expressed in *Tas1r3*+ cells. Type III and *Tas1r3*+ cells formed separate clusters (shown at the top), and markers of type II and type III cells also formed separate clusters (shown to the left).

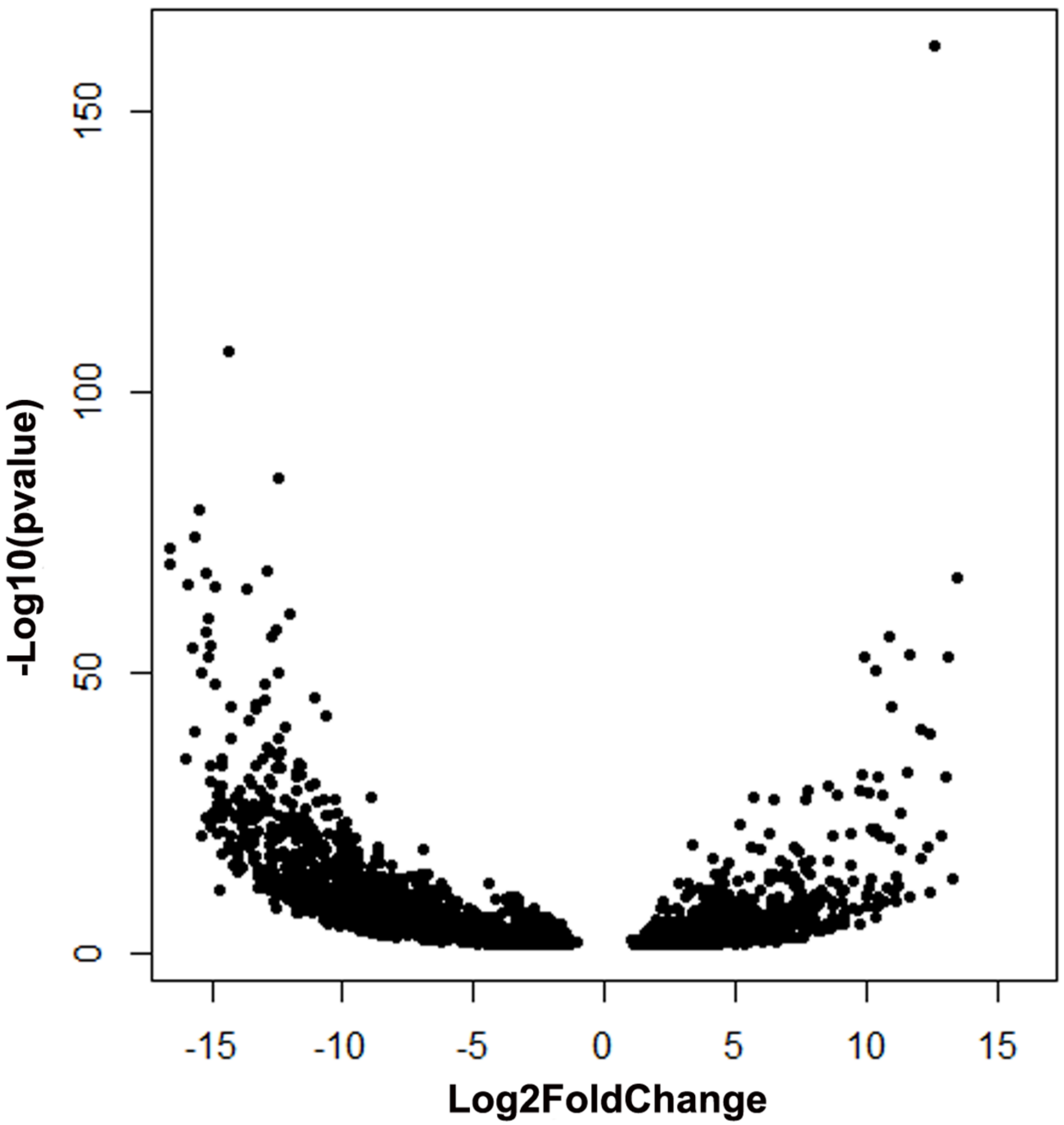

Supplementary Figure S4: Volcano plot of differentially expressed genes. Genes that are differentially expressed in type III (with positive Log2Fold change) and *Tas1r3*+ cells (with negative Log2Fold change) with absolute fold change ≥ 2, average expression ≥ 10 counts and false discovery rate (FDR) ≤ 0.05 identified by DESeq2 analysis are plotted.

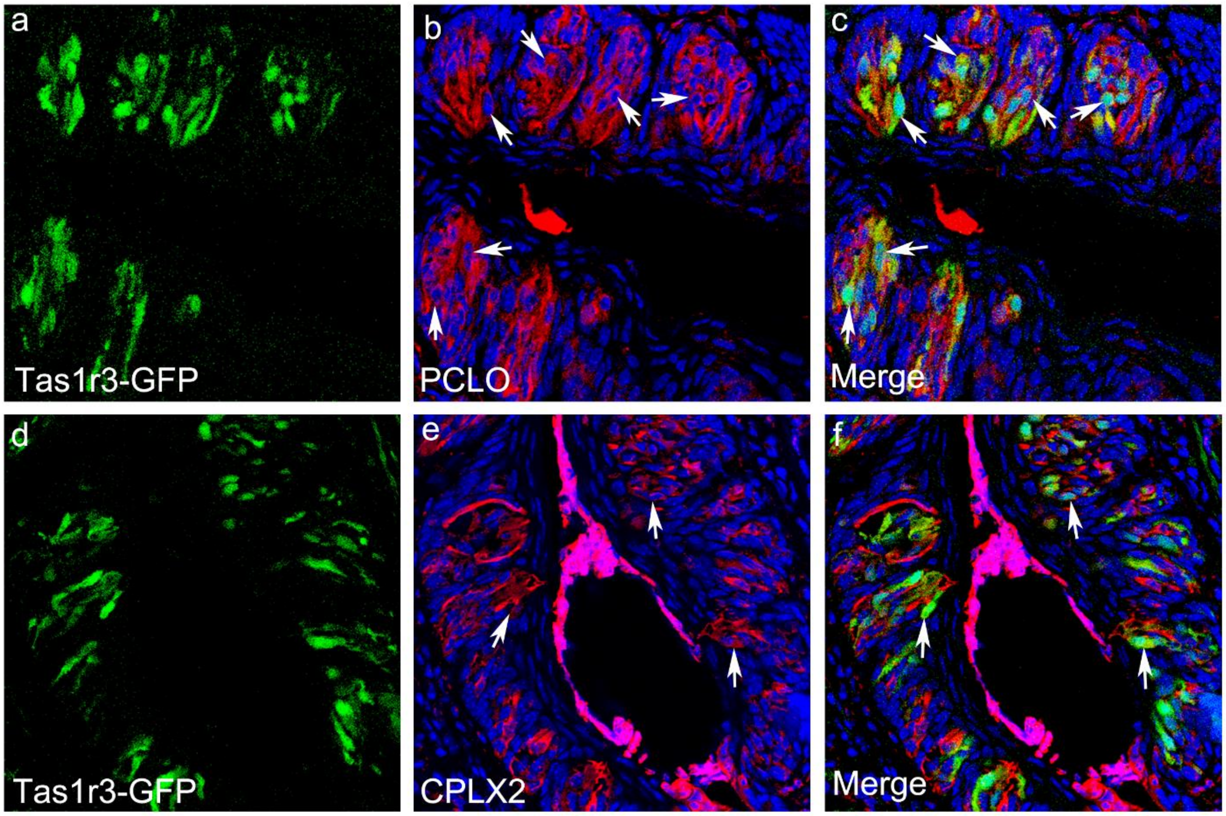

Supplementary Figure S5: Co-expression of Tas1r3-GFP (green, a, d) with synaptic vesicle components CPLX2 and PCLO (red, b, e). Merged images (c, f) show that CPLX2 is not co-expressed with Tas1r3-GFP, while PCLO is expressed in Tas1r3-GFP cells as well as in other taste cells not expressing Tas1r3-GFP. Arrows point to cells expressing PCLO or CPLX2 (b, e) and the same cells co-expressing Tas1r3-GFP (c, f). Scale bars: 20 µm.

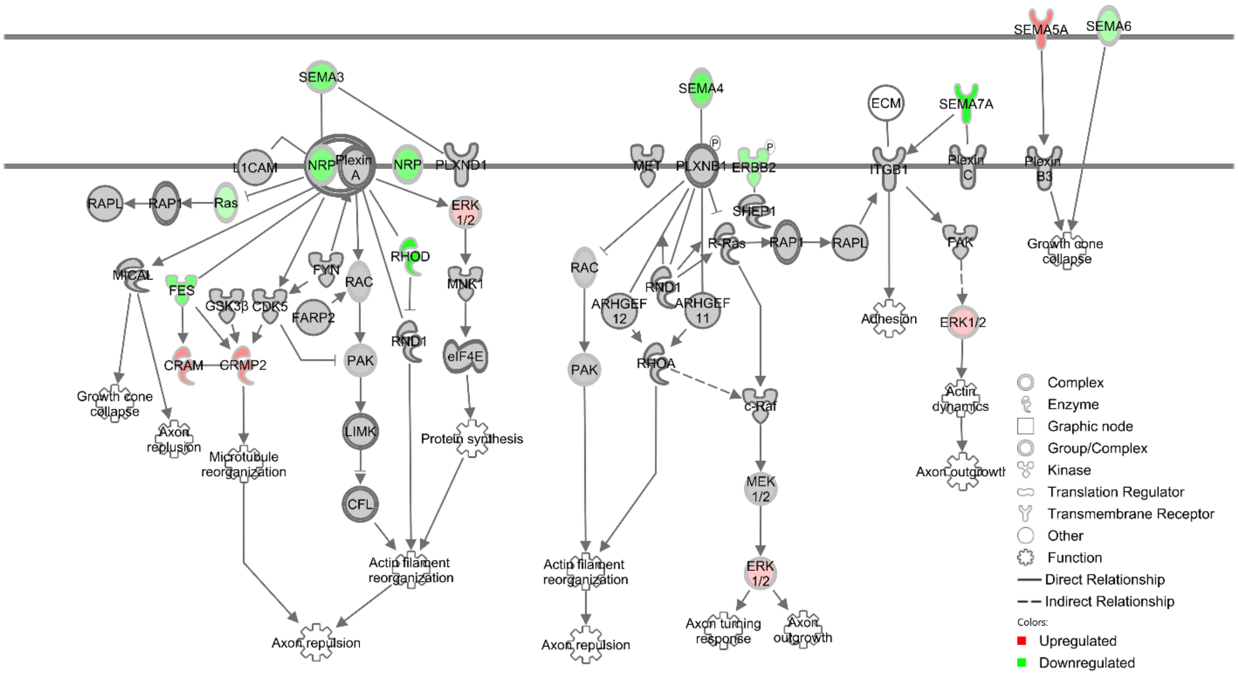

Supplementary Figure S6: The semaphorin signalling pathway. Genes which are up-regulated in type III cells with an FDR < 0.05 are shown in shades of red and those that are up-regulated in *Tas1r3+* cells are shown in green. Genes which did not pass the FDR cut-off are shown in grey.

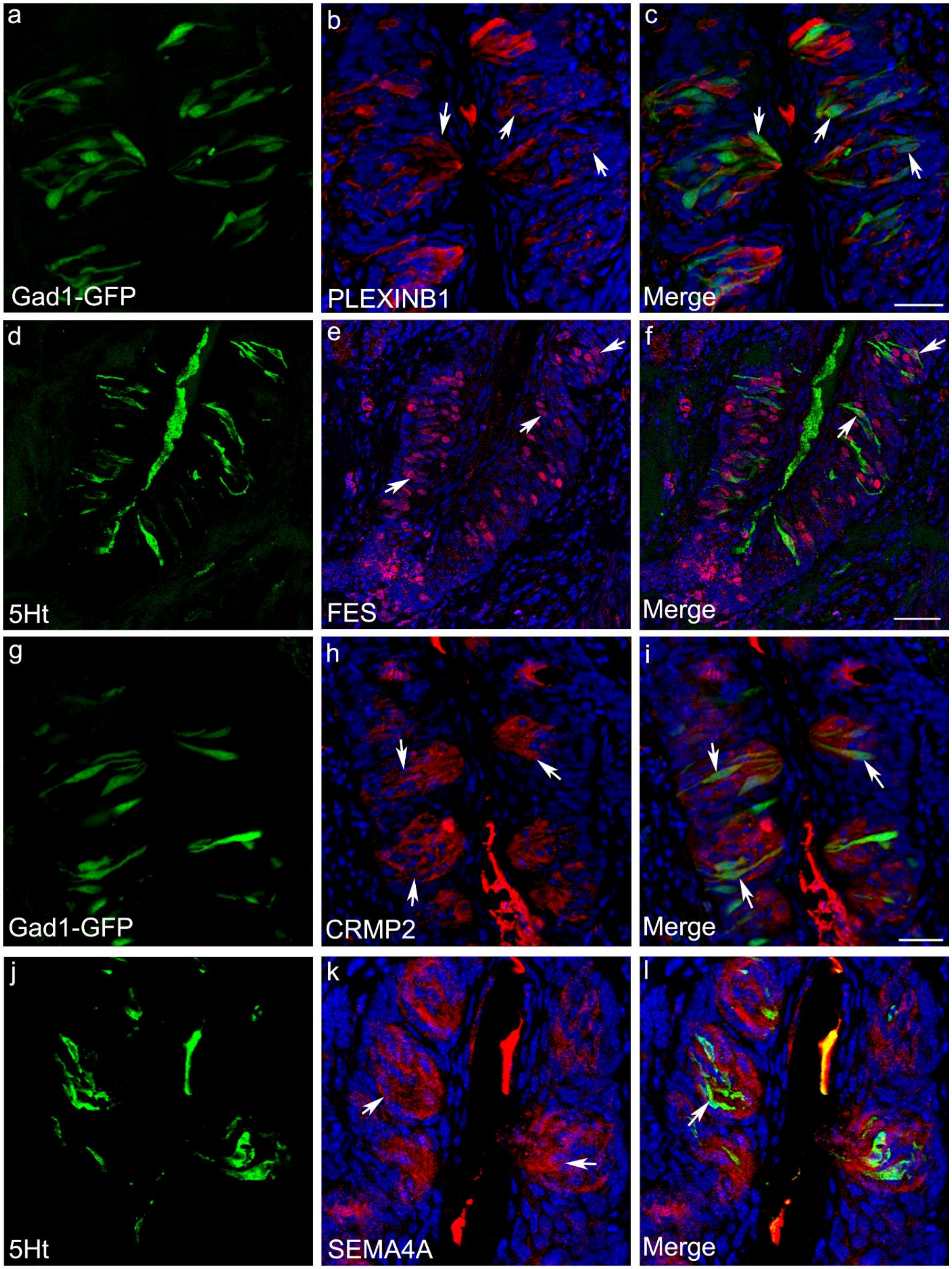
a
Gad1-GFP
b
PLEXINB1
c
Merge
d
5Ht
e
FES
f
Merge
g
Gad1-GFP
h
CRMP2
i
Merge
j
5Ht
k
SEMA4A
l
Merge

Supplementary Figure S7: Co-expression of semaphorin signalling pathway genes with the type III taste cell markers 5-HT or GAD1-GFP. GAD1-GFP (a,g) and 5HT (d, j) are shown in green, PLEXINB1, FES, CRMP2 and SEMA4A are shown in red (b,e,h and k),, along with the merged images (c, f, i, l). Arrows point to cells expressing PLEXINB1, CRMP2, FES or SEMA4A (b,e,h,k) and the same cells co-expressing type III cell markers (c,f,I,l). Scale bars: c, i, l =50 µm, f = 20 µm.

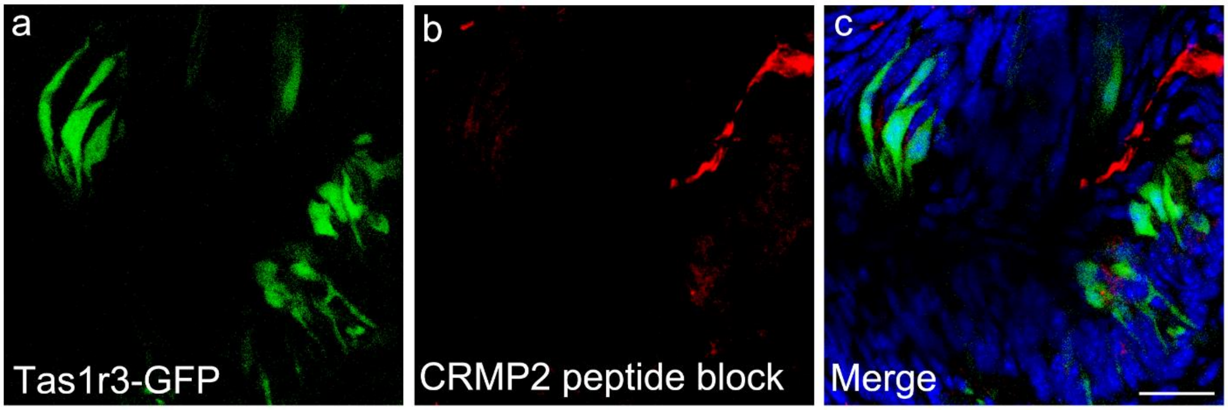

Supplementary Figure S8: Preincubation with CRMP2 peptide blocks CRMP2 antibody staining. Confocal images show: (a) Tas1r3-GFP fluorescence to identify taste cells, (b) CRMP2 antibody staining using antibody preincubated with the immunizing peptide and (c) the merged image. Scale bar = 30 µm.

Supplementary Table Legends

Supplementary Table S1. Summary of RNA-Seq results for individual *Tas1r3*+ (Tas_1-Tas_9) and type III (TIII_1-TIII_17) cells. Rows show data for each cell. Three libraries of low

quality (marked with asterisks) were excluded from further analysis. Columns show (left to right) labels of individual cell samples in format [Cell type identifier]_[Sample number] (e.g., Tas_1 = *Tas1r3*+ cell #1; TIII_1 = type III cell #1); the total number of raw reads; the number and proportion (relative to total reads) of reads that could be mapped to the mouse genome (Genome mapped reads); the number and proportion (relative to reads aligned to genome) of reads that map to more than one location in the genome (Multi-reads); the number and proportion (relative to reads aligned to genome) of reads mapping to exons of genes in the Gencode M4 set of annotations (Gene Mapped reads); the numbers of genes exceeding threshold levels (ranging from 1 to 50 normalized reads). Read counts were normalized using DESeq2. ND = not determined due to low quality of libraries.

Supplementary Table S2. Comparison of RNA-Seq results from *Tas1r3*+ and type III cells. Results show that the number of total genome-mapped and gene-mapped reads are not significantly different between *Tas1r3*+ and type III libraries, while that of multi mapping reads is significantly higher in *Tas1r3*+ libraries. Values represent means ± standard deviations, “Measurement” categories as described in Supplementary table S1 legend. Results of tests for significant differences between *Tas1r3*+ and type III cell libraries (Student’s two tailed t-tests with unequal variance) are listed under “p-Val (Tas1r3+ vs Type III)”. *Tas1r3*+ libraries express significantly higher numbers of genes than type III libraries above a range of thresholds from 1-50 mapped reads per gene, after normalization for sequencing depth.

Supplementary Table S3. Genes expressed in individual *Tas1r3*+ and type III taste cells. Individual genes are identified by their Ensemble Gene ID (“EnsembleGeneID”). The abbreviated gene name associated with each Ensemble Gene ID (“Gene Name”) and its full name (“Gene Description”) are also provided. We used DeSeq2 software to calculate average normalized

counts across all 23 cells ("Average Counts"); log2 fold change of average normalized counts in type III cells vs. those in *Tas1r3*+ cells ("log2FoldChange"); standard error of log2 fold change ("lfcSE"), Wald statistics ("stat" = Log2FoldChange/lfcSE, which DeSeq2 uses for calculating p-values); and p-values presented as un-adjusted ("pvalue") or adjusted for multiple comparisons using false discovery rate ("FDR"). Rows are sorted in descending order of p-values. FDR values for genes excluded by DESeq2 from multiple test correction using the independent filtering option are labelled NA. Counts from individual cell samples (labels as described in Supplementary table S1) are listed in columns from "TIII_1" (type III cell #1) through "Tas_9" (*Tas1r3*+ cell #9).

Supplementary Table S4. Euclidean distances between individual cells calculated based on patterns of RNA-Seq gene expression results. Euclidean distances were calculated in R and represented as a heat map in Fig 1b. Labels represent individual cell samples (see Supplementary table S1 legend).

Supplementary Table S5. Normalized expression (read numbers) of the three *Tas1r* genes in individual *Tas1r3*+ and type III cells. All three *Tas1r's* are expressed in most *Tas1r3*+ cells. Labels represent individual cell samples (see Supplementary table S1 legend).

Supplementary Table S6. The 3,466 genes differentially expressed in *Tas1r3*+ vs. type III cells. Criteria for labelling a gene as differentially expressed between *Tas1r3*+ and type III cells: absolute fold change ≥ 2, average expression ≥ 10, FDR-adjusted $p < 0.05$. This set of genes was used for generating the volcano plot shown in Supplementary Figure S4. Column labels as described in Supplementary Table S3 legend. General information on the known/predicted product of each gene obtained from the BioMart database is listed under the "Gene Type" heading.

Supplementary Table S7. The list of REVIGO- summarized Gene Ontology terms (GO terms) enriched in *Tas1r3*+ cells. The GO term accession numbers ("GO Term") from the Gene Ontology Consortium (www.geneontology.org) along with the associated function description ("Function") and function domain ("GO domain") are listed for GO terms enriched in *Tas1r3*+ cells. Frequency indicates the ratio of the number of genes associated with a corresponding GO term in our list of differentially expressed genes compared to the list of all genes in the background set. Uniqueness and dispensability are parameters from REVIGO analysis that rank GO terms according to how unique they are among other GO terms in their cluster. GO IDs with highest uniqueness and lowest dispensability scores are chosen as representative of a REVIGO cluster. Enrichment Score and enrichment p-value are from GO enrichment analysis.

Supplementary Table S8. The list of REVIGO- summarized GO IDs enriched in type III cells. Metrics as described in Supplementary Table S7 legend.

Supplementary Table S9. Cell death and survival functions and associated genes in type III cells compared with *Tas1r3*+ cells identified by IPA. A list of GO terms from the "Category" Cell Death and Survival that are differentially activated in type III vs *Tas1r3*+ cells. More detailed GO category descriptions can be found under "Diseases of Functions Annotation". P-value is calculated based on overlap between the set of focus genes and the set of all genes associated with the function in IPA, calculated using right tailed Fisher Exact test. Z score is calculated using the subset of these genes for which the directional effect on the function (increase or decrease) is known. IPA considers functions with an absolute Z score of 2 or above as significant. We have shown all functions with an absolute Z score of 1 or above. The number of molecules within each

GO grouping (“# Molecules”) and a complete list of these molecules (“Molecules”) are also provided.

Supplementary Table S10. Cell movement functions and associated genes in type III cells compared with *Tas1r3*+ cells identified by IPA. Same as Supplementary Table S9 but for GO terms associated with the category “Cellular Movement”.

Supplementary Table S11. Neuronal functions and associated genes in type III cells compared with Tas1r3+ cells identified by IPA. Same as Supplementary Table S9 but for GO terms associated with the category “Nervous System Development and Function”.

Supplementary Table S12. The top regulator effects networks generated by IPA using ‘cell movement’ and ‘nervous system development and function’ as filters. The top three regulator effect networks generated by IPA associated with the functions ‘cell movement’ and ‘nervous system development and function’ are shown. The consistency score (a measure of consistency of the observed relationship between members of the network in the dataset with that expected based on known relationship between genes), upstream ‘master’ regulators, intermediate regulators and the diseases and functions they regulate are shown. Top cellular movement networks upregulated in *Tas1r3*+ cells (consistency score 18.9) and in type III cells (consistency score 2.7) and the neuronal function related network upregulated in type III cells (consistency score 4.7) are shown. Networks 1 and 3 are also illustrated in Figures 3 and 4.

Supplementary Table S13. Synaptic components expressed in *Tas1r3*+ and type III cells above an average count of 20. The list of all synaptic components in the mouse genome were downloaded from Biomart (http://www.ensembl.org/biomart/) using the term GO:0045202 as filter.

Genes in this list represent a subset of the genes listed in Supplementary Table S3 (labels as described in Supplementary Table S3 legend). Genes with a low expression level (below average count of 20) were removed.

Supplementary Table S14. Semaphorin signalling pathway components expressed in *Tas1r3*+ and type III cells above an average count of 20. The list of all semaphorin signalling pathway components in the mouse genome were downloaded from Biomart using the term GO: 0071526 as filter. This table represents a subset of the genes listed in Supplementary Table S3 and were filtered as described in legend for Supplementary Table S13.

Supplementary Table S15. The list of synaptic component and semaphorin signalling pathway genes studied using immunohistochemistry. This table contains the data from Supplementary Table S3 for the genes examined using immunohistochemistry (Figures 5 and 6; Supplementary Figures S5, S7 and S8). See the legend for Supplementary Table S3 for description of the data in this table.
